# Subcellular RNA profiling links splicing and nuclear DICER1 to alternative cleavage and polyadenylation

**DOI:** 10.1101/gr.193995.115

**Published:** 2016-01

**Authors:** Jonathan Neve, Kaspar Burger, Wencheng Li, Mainul Hoque, Radhika Patel, Bin Tian, Monika Gullerova, Andre Furger

**Affiliations:** 1 Department of Biochemistry, University of Oxford, OX1 3QU, United Kingdom;; 2 Sir William Dunn School of Pathology, University of Oxford, OX1 3RE, United Kingdom;; 3 Department of Biochemistry and Molecular Biology, Rutgers New Jersey Medical School, Newark, New Jersey 07103, USA

## Abstract

Alternative cleavage and polyadenylation (APA) plays a crucial role in the regulation of gene expression across eukaryotes. Although APA is extensively studied, its regulation within cellular compartments and its physiological impact remains largely enigmatic. Here, we used a rigorous subcellular fractionation approach to compare APA profiles of cytoplasmic and nuclear RNA fractions from human cell lines. This approach allowed us to extract APA isoforms that are subjected to differential regulation and provided us with a platform to interrogate the molecular regulatory pathways that shape APA profiles in different subcellular locations. Here, we show that APA isoforms with shorter 3′ UTRs tend to be overrepresented in the cytoplasm and appear to be cell-type–specific events. Nuclear retention of longer APA isoforms occurs and is partly a result of incomplete splicing contributing to the observed cytoplasmic bias of transcripts with shorter 3′ UTRs. We demonstrate that the endoribonuclease III, DICER1, contributes to the establishment of subcellular APA profiles not only by expected cytoplasmic miRNA-mediated destabilization of APA mRNA isoforms, but also by affecting polyadenylation site choice.

Cleavage and polyadenylation (CPA) is a cotranscriptional pre-mRNA processing reaction that matures primary transcripts into functional mRNA molecules. In humans, more than half of all genes contain multiple CPA sites (Tian et al. 2005), and their alternative usage is widespread. Alternative cleavage and polyadenylation (APA) can lead to regulated synthesis of different mRNA isoforms. Although some transcripts generated by APA affect coding regions (CR-APA), the majority vary in the length of their 3′ untranslated region (3′ UTR, UTR-APA) (Supplemental Fig. S1). The relative positions of these CPA sites in the 3′ UTR of a gene and the sequence elements contained between them may result in the differential regulation of APA isoforms through RNA stability, translational efficiency, or subcellular localization (Sandberg et al. 2008; Weill et al. 2012).

The realization that APA occurs in most genes, and thereby potentially regulates widespread gene expression, triggered a flurry of investigations to annotate APA profiles in different tissues and cellular states (Ozsolak et al. 2010; Derti et al. 2012; Lin et al. 2012; You et al. 2015). RNA-seq–based global analysis extended the proportion of human genes that contain multiple CPA sites to ∼70% and demonstrated that APA not only occurs throughout tissues but may also be evolutionarily conserved (Tian et al. 2005; Derti et al. 2012; Miura et al. 2013).

Despite the recent focus on APA, underlying molecular mechanisms are hard to determine (Di Giammartino et al. 2011; Tian and Manley 2013). Nevertheless, several factors have been implicated in the global regulation of CPA site choice. These include the U1 snRNP (Berg et al. 2012), poly(A) binding protein (de Klerk et al. 2012; Jenal et al. 2012), the cytoplasmic cleavage and polyadenylation (CPEB1) factor (Bava et al. 2013), cleavage factor I (Martin et al. 2012; Masamha et al. 2014), FIP1L1 (Lackford et al. 2014), RBBP6 (Di Giammartino et al. 2014), the elongation rate of RNA polymerase II (RNAPII) (Martincic et al. 2009; Ji et al. 2011; Pinto et al. 2011), and several RNA-binding proteins (Al-Ahmadi et al. 2009; Mansfield and Keene 2012; Liu et al. 2013; Oktaba et al. 2015). Although these studies have demonstrated the prevalence of APA in eukaryotic cells, the extent to which APA alters RNA metabolism has recently come into question (Spies et al. 2013; Gupta et al. 2014; Neve and Furger 2014).

Most APA analysis has been performed using whole-cell RNA to assign APA profiles and regulatory models. Using this approach, it is difficult to discriminate between cause (choice of a CPA site at the point of cleavage) and effect of APA (altered stability or nuclear retention of particular isoforms). To address this, we compared APA profiles of mRNA isolated from whole-cell, nuclear, and cytoplasmic fractions. The rationale behind our approach is that primarily post-transcriptional regulatory mechanisms will act on cytoplasmic mRNA (Stoecklin et al. 2006; Valencia-Sanchez et al. 2006). Thus, APA isoforms that have different decay or nuclear/cytoplasmic export rates should differ in their relative isoform abundance in the cytoplasmic and nuclear fractions and so highlight APA isoforms that are regulated.

Using this approach, we identify differentially regulated APA isoforms, show that incomplete splicing can result in the nuclear retention of APA isoforms, and that CPA site selection can be influenced by nuclear DICER1.

## Results

### 3′-Region extraction and deep sequencing of subcellular RNA fractions

**Figure 1.**
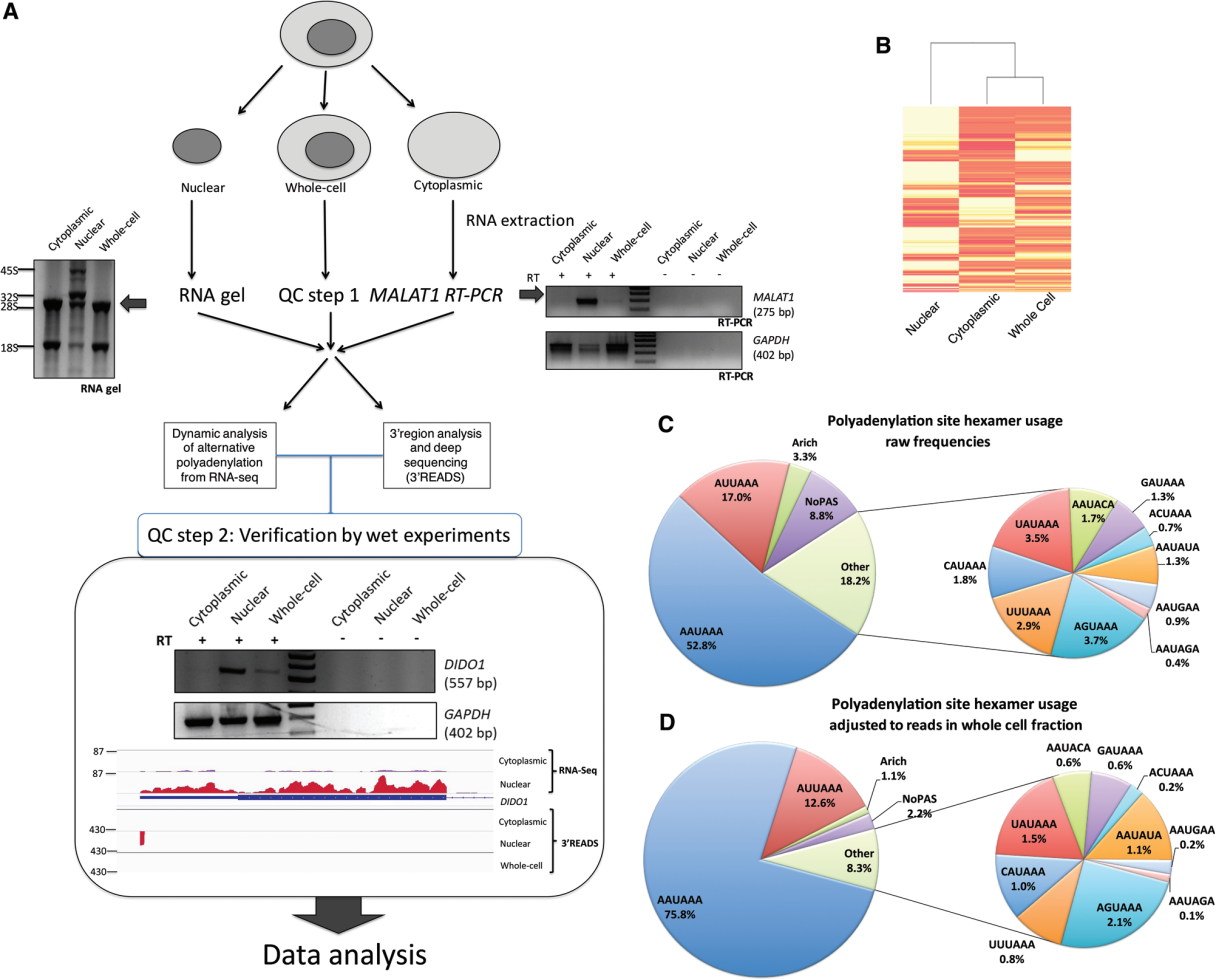
Subcellular fractionation-based APA analysis. (*A*) Schematic of the experimental approach showing the workflow and quality control steps. (*Left*) RNA gel and the enrichment of the precursor ribosomal RNAs in the nuclear fraction. (*Right*) RT-PCR of the nuclear marker *MALAT1* and the *GAPDH* loading control. (*Lower*) Verification of the deep-sequencing step, confirming the subcellular distribution of *DIDO1* as measured by 3′READS and RNA sequencing. (*B*) Heatmap and dendrogram plot showing similarity between subcellular fractions. (*C*) Polyadenylation signal (PAS) frequencies in the 0- to −40-nt region of all mapped cleavage and polyadenylation (CPA) sites. Here, only frequencies are taken into account, with all CPA sites considered equal, regardless of read number. (*D*) Read number–adjusted PAS frequencies, as in *C*, but with numbers reflecting reads over respective PAS in the whole-cell fraction of HEK293 cells.

We set out to determine APA profiles of purified nuclear and cytoplasmic RNA fractions (Fig. 1A) isolated from human embryonic kidney (HEK293) cells and HBL melanocytes. To quality control (QC) the purity of these fractions, we monitored the distribution of the highly abundant nuclear-retained long noncoding RNA (lncRNA), *MALAT1* (Hutchinson et al. 2007), and precursor ribosomal RNA species (pre-rRNA) (Fig. 1A, QC step 1; Supplemental Fig. S3). RNA fractions that passed QC were sequenced using the 3′READS method (Hoque et al. 2013) or standard poly(A)^+^ RNA-seq performed on an Ion Proton platform. As part of a second QC step, a minimum of 200-fold enrichment of the polyadenylated long noncoding RNA *XIST* (Jonkers et al. 2008) was set as a quantitative benchmark, and RT-PCR and Northern blotting of specific mRNAs served as additional controls (Fig. 1A, QC step 2; Supplemental Fig. S2).

### Genome analysis of CPA sites and APA isoform abundances

We first used the 3′READS protocol to establish accurate and quantitative APA profiles for each subcellular fraction from HEK293 cells. In each RNA fraction, 39,989 CPA sites were mapped, representing 14,633 genes. Of all mapped CPA sites, 76% were located in 3′ UTRs. The frequencies of the polyadenylation signals (PAS) observed (Fig. 1C) were similar to those reported in earlier studies (Wang et al. 2013). Importantly, when PAS frequencies were adjusted for read numbers per CPA site, the canonical hexamer frequencies increased to almost 90%, reflecting the strong link between CPA sites utilizing the canonical hexamer and high gene expression levels (Fig. 1D). This implies that although the frequency of noncanonical PAS is relatively high, they are associated with generally low expressed mRNA isoforms. Perhaps the lack of the conserved hexamer sequence may cause a loss in processing efficiency if it is not balanced by auxiliary elements (Dalziel et al. 2007, 2011) or strong downstream elements (Nunes et al. 2010).

Cross comparison of global mRNA isoform abundance between the three subcellular fractions shows that the whole-cell and cytoplasmic fractions of HEK293 cells are more similar to one another than either fraction is to the nuclear sample (Fig. 1B).

### Ten percent of mRNA APA isoforms in HEK293 cells are subject to significant differential regulation

We next examined the APA profiles in the three fractions in more detail and looked for APA events that are subjected to regulation. We restricted our analysis to the two most abundant 3′ UTR isoforms that showed a significant abundance change of >5% (Fisher's exact test, *P* = 0.01).

**Figure 2.**
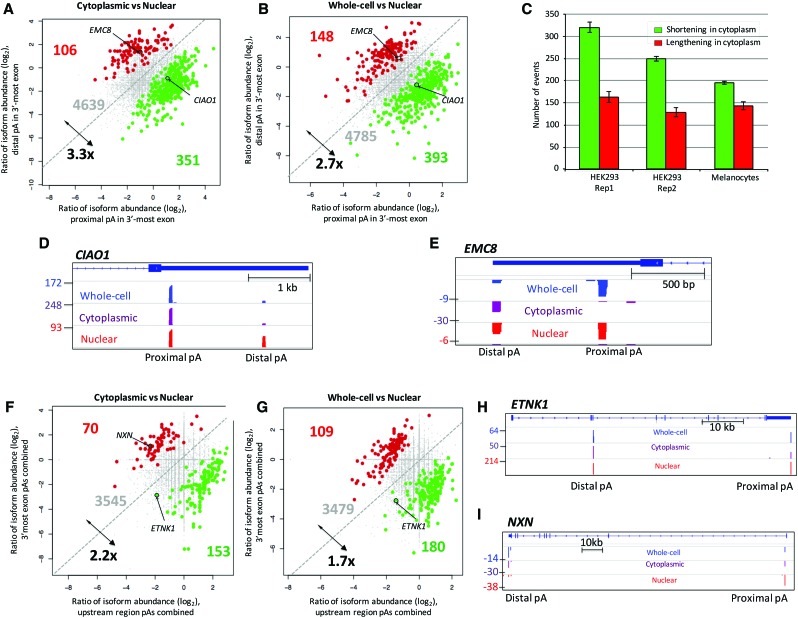
Differential representation of APA isoforms in subcellular RNA fractions using 3′READS. (*A*) Genes with 3′ UTR APA isoforms that differ significantly in relative representation of their two most highly expressed isoforms in the cytoplasm compared to the nucleus. Genes are highlighted when the shorter isoforms (green) or longer isoforms (red) have a significantly higher relative representation in the cytoplasm compared to the nucleus. Significantly regulated genes are those in which *P* ≤ 0.01 (Fisher's exact test) and the abundance change is >5%. (*B*) As in *A*, but comparing the whole-cell and nuclear APA profiles. (*C*) Number of genes showing significant difference in the cytoplasmic to nuclear fraction (C/N) ratio between proximal and distal CPAs in 3′ UTR based on the Significance Analysis of Alternative Polyadenylation (SAAP) method with FDR < 10%. The error bar is standard deviation based on 20 values. Green bars represent the number of genes showing a higher representation of proximal APA isoforms in the cytoplasmic fractions. Red bars represent the number of genes in which distal APA isoform is overrepresented in the cytoplasm. (*D*) Screenshot of *CIAO1* as an example of a gene showing overrepresentation of its shorter isoform in both the cytoplasmic and whole-cell fractions relative to the nucleus. Read numbers are shown on the *left*, and proximal and distal CPA sites (pA) are as indicated. (*E*) As in *D*. *EMC8* gene as an example showing overrepresentation of longer transcript in the cytoplasmic and whole-cell fractions. The positions of *EMC8* and *CIAO1* on the scatter plots are highlighted in *A* and *B*. (*F*) Genes with CR-APA, which significantly differ in their representation of upstream-region (intronic and exonic) APA isoforms relative to 3′-most-exon APA isoforms between the cytoplasmic and the nuclear fractions. Genes are highlighted when the upstream APA isoforms (green) or 3′ UTR isoforms (red) have a significantly higher relative representation in the cytoplasm compared to the nucleus. (*G*) As in *F*, but comparing whole-cell fraction relative to the nuclear fraction. (*H*) Screenshot of *ETNK1* as an example of a gene that shows an overrepresentation of the upstream APA isoform in both the cytoplasmic and whole-cell fractions relative to nucleus. (*I*) As in *H*, the *NXN* gene is an example of overrepresentation of the longer transcript in the cytoplasmic fraction compared to the nuclear pool. Positions of *ETNK1* and *NXN* on the scatter plots are highlighted.

Approximately 10% of all detected UTR-APA events in HEK293 and HBL melanocytes showed a significant change in relative isoform abundance between nuclear and cytoplasmic fractions (Fig. 2A–C). These isoforms displayed distinct characteristics, with the cytoplasmic and whole-cell fractions generally displaying a higher representation (approximately threefold) of transcript isoforms that use the promoter-proximal CPA sites (UTR shortening) relative to the nuclear fraction (Fig. 2A,B). Therefore, in genes with differentially regulated isoforms, shorter 3′ UTR isoforms are represented at higher levels in the cytoplasm as exemplified by *CIAO1* (Fig. 2D), consistent with the view that the 3′ UTR is generally repressive. However, there are a significant number of short 3′ UTRs that behave against this trend, for example, *EMC8* (Fig. 2E).

To address the reliability of these observations between biological replicates and different cell types, we compared the 3′READS analysis of cytoplasmic to nuclear fractions of two replicates of HEK293 cells and the neuronal crest–derived HBL melanocytes. For the robustness test, we used the Significance Analysis of Alternative Polyadenylation (SAAP) statistical test (FDR = 10%). SAAP compares the sample sets while controlling for sequencing depth (Li et al. 2015). As above, this analysis showed a consistent pattern whereby proximal APA isoforms were overrepresented in the cytoplasm relative to the nucleus (Fig. 2C).

We also identified around 3700 CR-APA events and again found ∼10% of them differed significantly between their abundance in the nuclear and the cytoplasmic fractions (Fig. 2F,G). Overrepresentation (approximately twofold) of the shorter isoforms in the cytoplasmic versus nuclear fraction as exemplified by *ETNK1* (Fig. 2H; Supplemental Fig. S8F) was again found for this type of APA. Only about 100 events were identified that showed an overrepresentation of the longer isoform in the cytoplasm such as *NXN* (Fig. 2I).

Thus, ∼10% of APA isoforms in resting HEK293 cells are subjected to significant differential regulation at the level of mRNA stability and/or nuclear retention.

### Overrepresentation of shorter APA isoforms in the cytoplasm is conserved between different cell lines but appears to be cell-type specific

APA analysis in subcellular fractions from HEK293 cells and melanocytes by 3′READS indicated a general overrepresentation of the shorter APA isoforms in the cytoplasm. To determine if this trend is true for different cell types, we used publicly available fractionated ENCODE RNA-seq data sets (Djebali et al. 2012). We only considered data sets that conformed to a minimum of a 20-fold enrichment of the strictly nuclear noncoding RNA *MALAT1* in the nuclear fractions relative to the equivalent cytoplasmic fractions.

To interrogate these data sets, we used the DaPars bioinformatics pipeline (Masamha et al. 2014). This algorithm compares the coverage across the annotated 3′ UTR of each gene to identify proximal CPA sites and quantifies the change in percentage distal CPA site usage index (ΔPDUI) between samples. We added an additional QC step to verify the validity of the CPA sites by filtering the predicted proximal CPA sites inferred by DaPars for “true” events, in which the predicted sites lie within 250 bp of a confirmed CPA site. The CPA site annotation used for this filter was from the APASdb, which mapped CPA sites throughout 22 human tissues (You et al. 2015).

**Figure 3.**
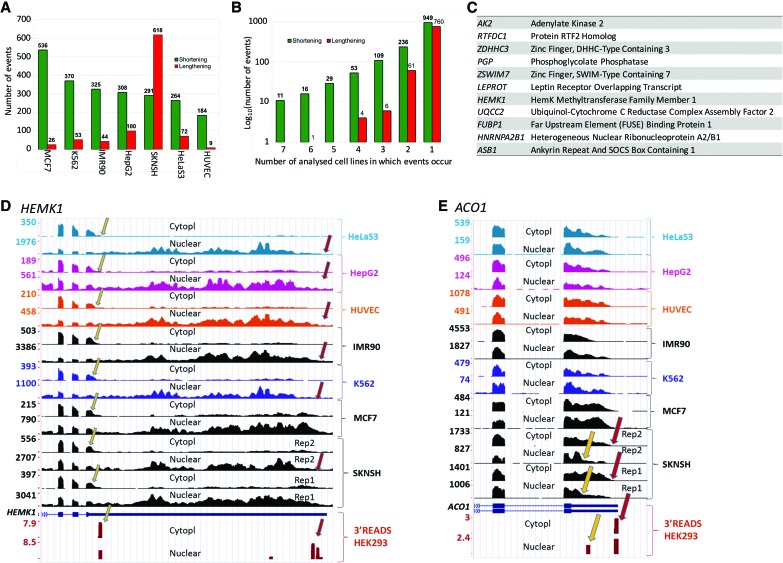
Differential representation of UTR-APA isoforms in subcellular RNA fractions using DaPars analysis. (*A*) Summary of the number of genes that show differential isoform representation in the subcellular fractions of seven different human cell lines using the DaPars algorithm. Green bars represent events in which there is a higher representation of the proximal APA isoform in the cytoplasm. Red bars show a higher representation of the distal APA isoform in the cytoplasm. (*B*) Analysis of the number of genes that show the same pattern of isoform representation across the different cell lines. Genes that show significant differential APA isoform representation using DaPars analysis were cross compared between cell lines. The number of events that occur in *x* number of cell lines were plotted on a log_10_ scale. Events in which the proximal APA isoform are overrepresented are shown in green, whereas events in which distal APA isoforms are overrepresented are shown in red. The absolute number of events is shown *above* each bar. (*C*) Identity of the 11 genes that show an overrepresentation of shorter APA isoforms in the cytoplasm in all seven cell lines. (*D*) Screenshot of the *HEMK1* gene showing an overrepresentation of the shorter APA isoform in the cytoplasm in all cell lines. The yellow arrows indicate proximal sites, and red arrows indicate distal CPA sites. The *bottom* two tracks show confirmation of CPA sites from our 3′READS data and also show the same distribution of proximal and distal isoforms in HEK293 cells. (*E*) As in *D*, but showing an overrepresentation of the longer APA isoform in cytoplasm of the SKNSH cell line.

Throughout the seven different cell lines analyzed by DaPars, we isolated 3180 separate genes that show differential APA isoform representation between cytoplasmic and nuclear fractions (Fig. 3A). As with our HEK293 and melanocytes data using 3′READS, APA events that showed differential profiles between the nuclear and cytoplasmic fractions represented ∼10% of all APA events (Supplemental Table 1). All cell lines analyzed, except for the neuroblastoma-derived SKNSH, confirmed the tendency to overrepresent shorter APA isoforms in the cytoplasm (Fig. 3A,D). SKNSH cells instead showed an overrepresentation of the longer APA isoform with extended 3′ UTRs in the cytoplasmic fraction compared to the nuclear fraction (Fig. 3A, SKNSH; Fig. 3E). Considering that whole-cell RNA reflects the cytoplasmic fraction (Fig. 1B), these longer isoforms in SKNSH cytoplasm may contribute to the characteristic feature of lengthening of 3′ UTRs observed in brain tissue (Miura et al. 2013).

Although short 3′ UTR-containing isoforms dominate in the cytoplasm of all non-neuronal tissue-derived cell lines, there is very little overlap between affected genes in these different cells (Fig. 3B). Only 11 APA cytoplasmic shortening events overlapped in all cell types analyzed (Fig. 3B,C), and only 10% were common to more than two cell lines. By far, the majority (75% of 1709) of APA shortening or lengthening events was found to be unique to the cell type (Fig. 3B). In order to confirm this was true cell-type specificity, rather than variation between isolations or within the analysis, the two biological replicates of MCF7 were analyzed individually and compared. This analysis showed an overlap of >70% of events that display differential representation of APA isoforms, confirming that the distinctive events seen are truly cell-type specific.

To explore the features in the alternative UTRs (aUTRs) that may contribute to the overrepresentation of the short APA isoforms in the cytoplasm, we interrogated the aUTRs of affected genes for potential regulatory 8-nt sequence motifs. We used the discriminative DNA motif discovery tool (DREME) (Bailey 2011) combined with the TOMTOM motif comparison search tool (Gupta et al. 2007). No motifs were found in the aUTRs of the 11 genes that showed consistent shortening through all cell lines. However, a number of motifs could be extracted when the aUTRs of shortening genes from each of the individual cell lines were interrogated separately. In particular, an A-rich sequence was present in genes that overrepresented short APA isoforms in cytoplasmic RNA extracted from HeLa, K562, IMR90, and HUVEC cells. This incorporates the AATAAA hexamer and so may represent alternative CPA sites located within the aUTRs (Supplemental Fig. S4). Only three of the remaining top motifs across all cell lines revealed sequences that show a similarity to known RNA-binding protein recognition sites (Supplemental Fig. S4).

From this analysis, we conclude that the general pattern of dominant shorter APA isoforms in the cytoplasm is consistent in non-neuronal cell types. However, the actual genes that undergo differential regulation of APA isoforms appear to be cell-type specific with no common motif or associated regulatory pathway.

### Gene-specific analysis of *CIAO1* APA isoforms suggests that intron retention can affect the nuclear cytoplasmic APA isoform distribution

**Figure 4.**
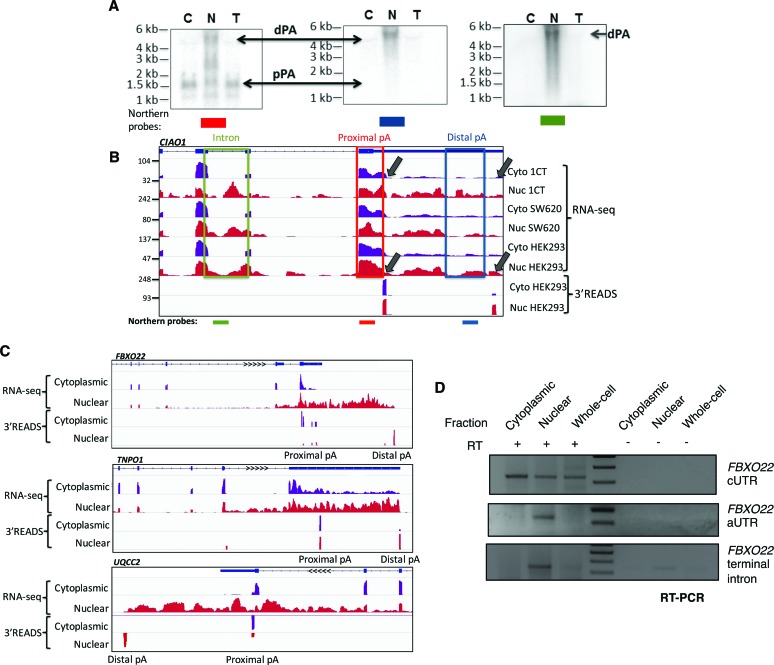
Overrepresentation of distal APA isoforms in the nucleus coupled to upstream intron retention. (*A*) Northern blot analysis of *CIAO1* APA isoforms in HEK293 subcellular fractions*.* Probes for constitutive UTR (*left*), alternative UTR (*middle*), and the upstream intron (*right*) were used to corroborate the 3′READS and RNA sequencing data. (*B*) RNA-seq and 3′READS tracks of subcellular fractions over the *CIAO1* gene. The cell line is indicated on the *right* of the tracks, and positioning of Northern probes used in *A* are shown *below*. (*C*) RNA-seq from HEK293 cells for three separate genes, all showing distal APA isoforms restricted to the nuclear fraction alongside an upstream intron retention event. For each event, proximal and distal CPA sites are shown as confirmed by 3′READS in the lower tracks. (*D*) RT-PCR confirming distribution of the two APA isoforms of *FBXO22* in HEK293 cells as well as the presence of the retained terminal intron in the nuclear poly(A)^+^ fraction.

As we were unable to identify any general feature that regulates subcellular APA profiles on a global scale, single gene analysis was carried out on the previously identified *CIAO1* APA isoforms (Fig. 2A,B,D). We first verified the nuclear cytoplasmic distribution profiles of the proximal (pPA) and distal APA (dPA) isoforms by Northern blotting by using a probe for the terminal exon to identify both APA isoforms (Fig. 4A,B, red probe) and a probe complementary to the aUTR sequence that specifically targets the distal *CIAO1* APA isoforms (Fig. 4A, middle; Fig. 4A,B, blue probe). The nuclear overrepresentation of the *CIAO1* distal isoform is unlikely to be due to cytoplasmic stability effects as judged from RNA stability analysis (Supplemental Fig. S5). Further analysis of RNA-seq data for *CIAO1*, (HEK293, 1CT, and SW620 colon cancer cells) revealed a relatively high number of reads (comparable to read numbers mapping to the flanking exons and the 3′ UTR) present in the region that corresponds to the *CIAO1* penultimate intron (Fig. 4B). Interestingly, similar observations were made when we interrogated HEK293 RNA-seq data for other nuclear-retained APA transcripts (Fig. 4C,D). Although the presence of reads mapped to intronic regions of nuclear RNA-seq samples are not unusual (Braunschweig et al. 2014), it nevertheless raised the possibility that the distal *CIAO1* APA isoform may represent a transcript that is incompletely spliced and so retained in the nucleus. Consistent with this hypothesis, use of an intron probe detected a transcript corresponding to the distal *CIAO1* APA isoform that was restricted to the nuclear fraction (Fig. 4A,B, green probe). This indicates that the nuclear-retained distal *CIAO1* isoform represents an incompletely spliced nuclear transcript and suggests that some APA profiles are the result of aberrant splicing that results in transcripts that are nuclear-retained.

### Some APA isoforms are regulated through miRNA-mediated degradation pathways

miRNA-targeting of aUTRs is widely considered a major mechanism for APA differential regulation (An et al. 2013). Furthermore, most miRNA-mediated regulation acts through degradation of targeted transcripts rather than translational inhibition (Guo et al. 2010).

**Figure 5.**
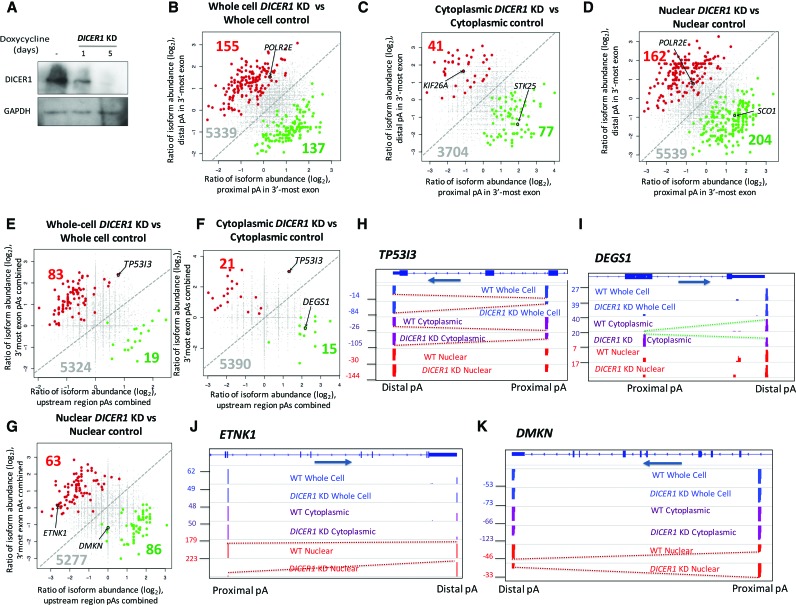
miRNA-based stability plays a minimal role in the differential regulation of APA isoforms. (*A*) Western blot showing depletion of endogenous DICER1 after a 5-d induction with doxycycline. GAPDH was used as a loading control. (*B*) Genes with 3′ UTR-APA that show either a significant shortening (green) or lengthening (red) of 3′ UTRs upon *DICER1* KD when comparing whole-cell fractions. (*C*,*D*) As in *B*, but comparing cytoplasmic fractions (*C*) and nuclear fractions (*D*) in *DICER1* KD cells relative to the equivalent control fractions. Screenshots of genes highlighted in scatter plots in *B*, *C*, and *D* are shown in Supplemental Figure 6. (*E*,*F*,*G*) As in *B*, *C*, and *D* but showing CR-APA rather than UTR-APA. (*H*) Screenshot of *TP53I3*, showing lengthening in the *DICER1* KD cells when comparing both the cytoplasmic and whole-cell fractions. (*I*,*J*,*K*) As in *H*, showing *DEGS1, ETNK1*, and *DMKN*, respectively. *DEGS1* shows shortening to the upstream CPA site compared to cytoplasmic fractions upon *DICER1* KD. *ETNK1* and *DMKN* show lengthening and shortening, respectively, when comparing the nuclear fractions of the *DICER1* KD cells to the control cells. The positions of these genes on the scatter plots are highlighted.

To quantify miRNA-mediated degradation of APA isoforms in the cytoplasm of HEK293 cells, an inducible *DICER1* knockdown (*DICER1* KD) HEK293 cell line was used (Schmitter et al. 2006). Cells were grown in the presence or absence of doxycycline, to induce a short hairpin RNA (shRNA) that targets *DICER1* mRNA. Following a five day induction period, DICER1 protein was reduced by ∼95% (Fig. 5A). This time point was chosen to avoid secondary effects (Schmitter et al. 2006), but to exceed the long half-lives of some miRNAs (Bail et al. 2010; Gantier et al. 2011).

Comparison of the whole-cell *DICER1* KD with whole-cell control RNA revealed that 300 APA isoforms (5% of total APA) were significantly affected upon loss of DICER1 (Fig. 5B). To confirm that the effects on whole-cell APA profiles observed in the *DICER1* KD cells are due to the expected miRNA-mediated destabilization of transcripts in the cytoplasm, we subjected subcellular fractionations of control and knockdown cells to 3′READS analysis. We identified around 100 or 3% of APA isoform frequencies that changed significantly between the cytoplasmic fractions of *DICER1* wild-type (WT) and KD cells (Fig. 5C). Of those that changed, 41 genes showed an increase in distal CPA site usage when DICER1 is depleted. This cohort is exemplified by *KIF26A* (Fig. 5C; Supplemental Fig. S6A), which harbors predicted miRNA binding sites in its aUTR, indicating that its distal APA isoform is specifically targeted and destabilized by a miRNA. As represented by *STK25* (Fig. 5C; Supplemental Fig. S6B), around twice as many genes were extracted in which the relative frequency of the APA isoforms with short 3′ UTRs increased upon *DICER1* KD (Fig. 5C).

From this analysis, we conclude that ∼3% of all cytoplasmic APA events are subjected to differential miRNA-mediated regulation, and miRNA-mediated degradation of APA transcripts targets proximal and distal APA isoforms.

### *DICER1* knockdown has a profound effect on the nuclear APA profiles

We next analyzed the effect of *DICER1* KD cells on APA in the nuclear fraction and surprisingly found a greater (6%) change than for the cytoplasmic APA fraction. In the nucleus, the knockdown of *DICER1* caused both 3′ UTR lengthening as exemplified by *POLR2E* (Fig. 5D; Supplemental Fig. S6C) and 3′ UTR shortening represented by *SCO1* (Fig. 5D; Supplemental Fig. S6D).

The selective effect of *DICER1* knockdown on nuclear UTR-APA profiles was unexpected. We therefore also tested whether the regulation of APA isoforms through DICER1-dependent mechanism also affects CR-APA. The usage of CPA sites that are located in upstream introns or exons with the sum of all reads in the 3′ UTR of the same gene was tested. Less than 100 CR-APA isoforms were identified in the whole-cell fractions that showed a significant change in CPA site usage upon *DICER1* knockdown (Fig. 5E). By analyzing the cytoplasmic fractions, we identified 36 CR-APA isoforms that change significantly in response to depletion of DICER1 in the cytoplasm (Fig. 5F). Similar to UTR-APA, DICER1 depletion causes a change of frequencies of both distal APA isoforms, illustrated by *TP53I3* (Fig. 5H) and of proximal APA isoforms, as demonstrated by *DEGS1* (Fig. 5I). Interestingly, as has been observed with the UTR-APA analysis, the significant changes of CR-APA isoform abundances upon DICER1 depletion were more profound when the nuclear fractions of *DICER1* knockdown and control cells were compared (Fig. 5G). Relative to the RNA fractions from control cells, DICER1-depleted RNA nuclear fractions had chiefly an equal number of cases, where distal CPA site usage (*ETNK1* in Fig. 5J; Supplemental Fig. 8E) or proximal CPA site usage (shortening, as exemplified by *DMKN* in Fig. 5K) increased.

Overall, we show that some APA is subject to post-transcriptional miRNA-dependent regulation, and the effects of DICER1 depletion are not restricted to cytoplasmic but extend to the nuclear APA fraction. This suggests direct and/or indirect effects of DICER1 on CPA site choice.

### DICER1 associates with the *ETNK1* proximal CPA site and modifies the surrounding chromatin

**Figure 6.**
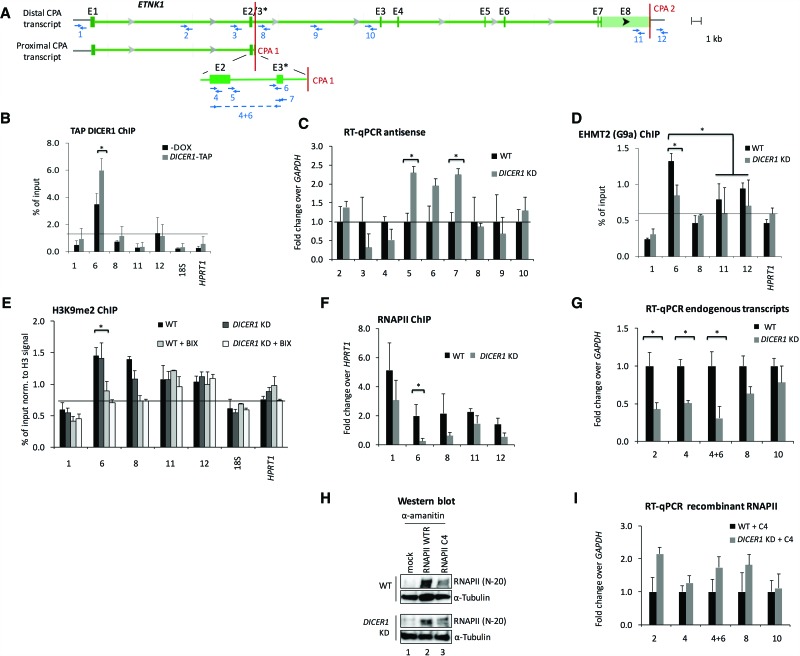
DICER1 regulates proximal CPA site usage at the *ETNK1* locus. (*A*) Schematic showing positions of ChIP amplicons and RT-qPCR probes (1–12) on the human ethanolamine kinase 1 (*ETNK1*) locus around proximal and distal CPA sites 1 and 2 (CPA 1 and 2): (E3*) alternative exon 3. (*B*) ChIP analysis of TAP-tagged DICER1 occupancy over *ETNK1*. Primer pairs analyze the region surrounding *ETNK1* CPA sites: (6, 8) intronic CPA 1; (11, 12) canonical CPA 2. Values from noninduced and *DICER1* KD HEK293 cells are shown as a percentage of input. Mean values and standard deviations were calculated from at least three biological replicates: (*) *P* < 0.05 by two-tailed Student's *t*-test. (*C*) Antisense RNA levels around *ETNK1* at CPA 1. Primer pairs specific to intronic and exonic antisense transcripts were used to determine relative changes in antisense RNA levels upon *DICER1* KD by RT-qPCR. (*D*,*E*) ChIP analysis as in *B* of EHMT2 histone methyltransferase (*D*) and histone 3 lysine 9 dimethylation (H3K9me2) (*E*): (18S) 18S ribosomal RNA; (BIX) BIX-01294 trihydrochloride hydrate. (*F*) RNA polymerase II (RNAPII) occupancy at human ethanolamine kinase 1 (*ETNK1*) CPA sites. The percentage of input values from noninduced and *DICER1* KD HEK293 cells are shown as fold change over *HPRT1* signals. (*G*,*I*) Levels of intronic and exonic sense transcripts around *ETNK1* CPA 1 from endogenous RNAPII transcription (*G*) and upon reconstitution of transcription with recombinant RNAPII construct C4 (*I*). Signals from noninduced and *DICER1* KD HEK293 cells are shown as fold over wild type and normalized to *GAPDH* sense transcript levels. Mean values and standard deviations were calculated from at least three biological replicates: (*) *P* < 0.05 by two-tailed Student's *t*-test. (*H*) Western blot showing expression levels of recombinant, α-amanitin resistant RNAPII mutant WTR (recombinant wild type) and C4 (slow elongating mutant) in wild-type and *DICER1* KD HEK293 cells: (mock) empty vector.

The observed DICER1-dependent changes to the nuclear APA fraction can be either direct or indirect. Indirect effects could be due to DICER1-dependent changes to the expression of key components of the cleavage and polyadenylation machinery. However, depletion of DICER1 has only a modest impact on their expression (Supplemental Fig. S7), suggesting that the nuclear effects may be direct. Several recent papers have described nuclear functions of DICER1 (Sinkkonen et al. 2010; Doyle et al. 2013; Skourti-Stathaki et al. 2014; White et al. 2014). To explore the possibility of a direct role of DICER1 in CPA site choice in *ETNK1*, we performed ChIP experiments using amplicons covering critical regions in the *ETNK1* gene (Fig. 6A; Supplemental Fig. S8A) that showed a distinct loss of the proximal APA isoform in the nucleus in the absence of DICER1 (cf. WT nuclear and *DICER1* KD nuclear) (Fig. 5J). Using an antibody targeting endogenous DICER1 (Supplemental Fig. S8C), we detected an enrichment of DICER1 at amplicon 6, which is in close vicinity of the proximal CPA site. This signal was significantly reduced in DICER1-depleted cells (WT versus *DICER1* KD) (Supplemental Fig. S8C). Since ChIP signal intensity with the antibody targeting endogenous DICER1 was low and to further validate this initial finding, we established a HEK293 cell line with an inducible integrated TAP-tagged *DICER1* allele. This cell line also expresses an integrated doxycycline-inducible shRNA that specifically targets endogenous but not TAP-tagged *DICER1* (Supplemental Fig. S8B). ChIP analysis using Sepharose IgG beads in doxycycline-induced cells enhanced the signal to noise ratio (cf. amplicon 6 to 18S and *HPRT1* amplicons) (Fig. 6B) and corroborated the significant enrichment of DICER1 signals in amplicon 6 in *ETNK1* (Fig. 6A,B). In contrast, no significant changes in DICER1 levels were observed +/− *DICER1* KD between amplicons covering the critical regions in *DMKN* and *SCO1* (Supplemental Fig. S9A–C), where *DICER1* KD resulted in a preference of the distal CPA sites. This suggests that DICER1 may specifically associate with the proximal CPA sites. Since the recruitment of DICER1 to specific chromosomal regions was recently linked to the formation of double-stranded RNA (Skourti-Stathaki et al. 2014; White et al. 2014), we used a qRT-PCR–based approach to detect potential antisense transcripts that would overlap the region of the proximal CPA site and could explain the accumulation of DICER1 in this region. Interestingly, upon DICER1 depletion, antisense transcripts accumulated above background signals in the region spanning the *ETNK1* proximal CPA site (Fig. 6C). Furthermore, the DICER1 signal was lost in the proximal CPA region when the chromatin was subjected to RNase treatment prior to immunoprecipitation (Supplemental Fig. S8G). These antisense transcripts may thus have the potential to hybridize to *ETNK1* sense transcripts and could lead to dsRNA formation, the natural substrate of nuclear DICER1 (Skourti-Stathaki et al. 2014; White et al. 2014). Interestingly, no such antisense transcript was detected in the *DMKN* locus (Supplemental Fig. S9D).

Nuclear DICER1 processes long dsRNA substrates into small interfering RNAs, which leads to the formation of repressive heterochromatin marks (H3K9me2) formed by the action of the histone methyltransferase EHMT2 (also known as G9a) (White et al. 2014). To determine if similar events are replicated at the *ETNK1* proximal CPA sites, we used a ChIP-based approach to monitor the presence of the histone methyltransferase EHMT2 and its characteristic H3K9me2 modification over this region. Strikingly, similar to the DICER1 ChIP profile, we detected a positive EHMT2 methyltransferase ChIP signal in amplicon 6 of *ETNK1*, which was reduced upon DICER1 depletion (Fig. 6D, amplicon 6). We also detect significant enrichment for H3K9me2 signal over the same amplicon 6 (Fig. 6E), and a small reduction of this mark was seen in *DICER1* KD cells in amplicon 8 (Fig. 6E). We did not detect any significant levels of H3K9me3, which is a heterochromatin mark specific for centromeric regions in the *ETNK1* amplicons (Supplemental Fig. S8D). To further address potential effects on the H3K9me2 profiles, we treated WT and *DICER1* KD cells with the compound BIX-01294, a chemical inhibitor of the EHMT2 methyltransferase (Supplemental Fig. S10). Exposure to BIX resulted in a reduction of the H3K9me2 ChIP signals at regions surrounding the *ETNK1* proximal but not distal CPA site (cf. amplicons 6 and 8 with 1, 11, and 12) (Fig. 6E). The reduction of signal was more pronounced when BIX treatment was performed on cells with reduced levels of DICER1 (cf. WT with WT + BIX and *DICER1* KD + BIX) (Fig. 6E). Overall, our results suggest that the H3K9me2 mark surrounding the proximal CPA site (amplicons 6 and 8) may be EHMT2 and DICER1 dependent.

To further elucidate the mechanism that controls proximal CPA site usage, we determined the RNAPII profile and nascent transcript levels over the *ETNK1* locus. We detected a significant decrease in RNAPII levels around the proximal CPA site in the absence of DICER1 (Fig. 6F, amplicon 6). In contrast, RNAPII levels are not significantly decreased in DICER1-depleted cells at amplicons over *DMKN* (Supplemental Fig. S11A) and *SCO1* (Supplemental Fig. S11B) loci.

The decreased RNAPII levels observed in the regions surrounding the proximal CPA site of *ETNK1* (Fig. 6F) could reflect generally lower RNAPII levels when DICER1 is depleted. Alternatively and based on the results presented above, it is possible that depletion of DICER1 enforces a more “relaxed” heterochromatin conformation, which may enable RNAPII to progress faster through this region. To test these two possibilities, we used qRT-PCR using specific primers to detect *ETNK1* nascent transcripts in WT and *DICER1* KD cells (Fig. 6G). Amplicons 2 and 4 reflect the transcript levels of both shorter and longer *ETNK1* nascent isoforms, amplicons 8 and 10 represent the levels of only the longer *ETNK1* nascent transcript, and amplicon 4 + 6 represents levels of solely the proximal *ETNK1* transcript (Fig. 6A). The qRT-PCR revealed that the levels of the amplicons 2 and 4 and 4 + 6 are significantly decreased in *DICER1* KD cells. This is in contrast to the levels observed over amplicons 8 and 10, which remain similar in WT and *DICER1* KD cells (Fig. 6G). To further explore the effect on isoform expression under conditions of a slow moving polymerase, we transfected WT and *DICER1* KD cells with plasmids carrying the doxycycline-inducible α-amanitin resistant largest subunit of RNAPII. Two plasmids that encode either for a wild-type subunit or the RNAPII C4 mutant subunit that has the characteristic of a “slow polymerase” (de la Mata et al. 2003) were transfected into HEK293 wild-type and *DICER1* KD cells, and expression of the subunits was confirmed by Western blot (Fig. 6H). Endogenous RNA polymerase activity was suppressed by exposing the cells to α-amanitin. Total RNA was subsequently isolated from these cells and subjected to qRT-PCR analysis. Interestingly, the levels of *ETNK1* nascent transcripts over amplicons 2,4, and 4 + 6 in *DICER1* KD compared to wild-type cells are not reduced but rather slightly elevated (Fig. 6I). This suggests that a slow moving RNAPII can rescue the *DICER1* KD phenotype and maintain the recognition of the proximal CPA site even in the absence of DICER1. A possible explanation for the rescue could be that the nature of the slow moving polymerase prevents its acceleration through this region despite the more relaxed chromatin environment caused by the depletion of DICER1.

Taken together, these results suggest that DICER1 and EHMT2 contribute to shaping the chromatin landscape surrounding the proximal CPA site in *ETNK1*, forcing RNA polymerase to slow down, which in turn may favor proximal PAS recognition. How far reaching this process is remains to be explored.

## Discussion

Our comparison between cytoplasmic and nuclear APA isoform abundance throughout nine human cell lines reveals that although ∼70% of genes use APA, the majority of these APA events do not show significant differences in relative abundance between the subcellular fractions (Figs. 2, 3). Furthermore, isoforms that do show significant differences appear to be tissue-specific events. Our results suggest that the vast majority of APA events detected have little regulatory impact at the level of RNA stability and/or nuclear cytoplasmic export rates.

Several studies have found a correlation between 3′ UTR length and predicted miRNA target sites, suggesting that this is a major pathway for the differential regulation of mRNA APA isoforms (Sandberg et al. 2008; Ji and Tian 2009; Liaw et al. 2013; Miura et al. 2013; Tian and Manley 2013). Indeed, our use of the *DICER1* KD cells and subsequent APA analysis with the fractionated RNA pools does reveal a number of APA isoforms that are likely to be regulated by miRNAs. However contrary to expectation, many of these 3′ UTR isoforms show shortening rather than lengthening upon *DICER1* KD (Fig. 5). Although unexpected, this may reflect the occlusion of miRNA sites by secondary structures formed in extended 3′ UTRs (Ameres et al. 2007).

We show that the vast majority of APA isoforms are not differentially exported from the nucleus nor do they differ in mRNA stability. In addition, although aUTRs have been shown to control membrane localization of their resulting proteins at an individual gene level (Berkovits and Mayr 2015), the global impact of aUTRs on translation efficiency appears limited (Gruber et al. 2014). This raises the possibility that many APA events in resting cells may be inconsequential and not specifically controlled events. Such a “random” nature of APA has been previously proposed when analyzing the conservation of APA throughout evolution (Ara et al. 2006). Perhaps in many cases, APA is the result of a fail-safe mechanism to ensure that fully transcribed and spliced transcripts obtain a mature 3′ end (Proudfoot 2011). It is possible that each CPA site has a given frequency of usage determined by its immediate and wider sequence composition (Nunes et al. 2010). In many transcripts, where the first CPA site escapes recognition, a downstream site will be captured extending the 3′ UTR without any major consequences for its metabolism or translational output. This would also fit with the proximal CPA sites generally having lower consensus sequences (Martin et al. 2012).

Although many APA events may have little physiological impact, an important subset of APA isoforms is undoubtedly critical for both establishing tissue-specific gene expression signatures and driving cell differentiation, growth, and also disease phenotypes (Masamha et al. 2014; Berkovits and Mayr 2015). Identifying which APA isoforms are regulatory will be imperative to understand the physiological impact of APA. The cellular fractionation approach described here provides a method of identifying regulatory APA events in an unperturbed system; and using this method, we identified two mechanisms that can shape the nuclear APA profile.

Firstly, as exemplified by *CIAO1*, we identified incomplete splicing results in differential representation of the individual APA isoforms in the cytoplasm and nucleus (Fig. 4). Interestingly, intron retention is linked to usage of the distal CPA site, which would support the aforementioned fail-safe hypothesis in which the distal CPA site is used when the proximal CPA site is not adequately recognized—most likely due to the perturbation of the well-documented interconnection between splicing and 3′ end processing (Proudfoot et al. 2002). The mechanism of transcript retention is likely to involve the presence of splice factors that are associated with the incompletely spliced distal APA isoform (Takemura et al. 2011).

Secondly, our analysis of nuclear and cytoplasmic APA profiles under conditions in which DICER1 is depleted identifies DICER1 as an unexpected regulator of APA at the point of CPA site choice (Fig. 6). There is mounting evidence that DICER1 is present and functional in the nucleus (Sinkkonen et al. 2010; Skourti-Stathaki et al. 2014; White et al. 2014). In this context, a role for nuclear DICER1 in APA is plausible. Although we cannot exclude that the shifts in nuclear APA profiles observed upon DICER1 depletion are partly the result of secondary effects, our analysis of the *ETNK1* locus provides evidence in support of a direct involvement of nuclear DICER1 in CPA site selection. Our ChIP-based analysis not only shows that both DICER1 and the EHMT2 methyltransferase associate with chromosomal regions close to a regulated CPA site (Fig. 6B,D), but these observations are complemented by the H3K9me2 profiles that establish a distinct chromatin landscape surrounding the proximal CPA site (Fig. 6E). This is the likely mechanism by which the speed of RNAPII is regulated over this region. Thus, DICER1 may promote heterochromatin formation in the regions surrounding proximal CPA sites of certain genes (Fig. 6) and so regulate the speed of transcribing RNAPII. This in turn will enhance recognition of the proximal CPA site. The concept that a slow or paused RNAPII can aid PAS recognition has previously been demonstrated by in vitro transcription experiments (Yonaha and Proudfoot 1999).

## Methods

### Subcellular RNA fractionation

The subcellular fractionation protocol used was adapted from Topisirovic et al. (2003). A detailed description of the technique can be found in the Supplemental Material.

### RNA analysis

Northern blotting, ChIP and RT-PCR protocols, and oligonucleotides used are detailed in the Supplemental Material.

### 3′READS protocol and analysis of 3′READS data

cDNA sequencing libraries were generated, sequenced, and analyzed as previously described in Hoque et al. (2013) and the Supplemental Material.

### Dynamic analysis of APA from RNA-seq

DaPars analysis was performed as described in the original paper (Masamha et al. 2014) by using the code available at https://code.google.com/p/dapars with default settings. The data sets used for analysis are as previously published (Djebali et al. 2012) and available at http://genome.ucsc.edu/ENCODE/dataMatrix/encodeDataMatrixHuman.html.

### Motif analysis

aUTRs were extracted, and the DREME motif analysis tool with default parameters was used to identify overrepresented motifs using a scrambled reference. Motifs were then submitted to the TOMTOM motif comparison tool.

## Data access

The sequencing data from this study have been submitted to the NCBI Gene Expression Omnibus (GEO; http://www.ncbi.nlm.nih.gov/geo/) under accession number GSE68671.

## Supplementary Material

Supplemental Material
