# Supplementary material for: Subcellular RNA profiling links splicing and nuclear DICER1 to alternative cleavage and polyadenylation
Source: Genome Res. 2016 Jan;26(1):24–35. doi: 10.1101/gr.193995.115 (PMC4691748; doi:10.1101/gr.193995.115)
Supplement: Supplemental Material [file supp_26_1_24__index.html]

Subcellular RNA profiling links splicing and nuclear DICER1 to alternative cleavage and polyadenylation — Subcellular RNA profiling links splicing and nuclear DICER1 to alternative cleavage and polyadenylation — Supplemental Material 

# Subcellular RNA profiling links splicing and nuclear DICER1 to alternative cleavage and polyadenylation

## Supplemental Material

**Files in this Data Supplement:**

- Supplemental Figures.pdf
- Supplemental Information.docx
- Supplemental Table 1.xls
