# Supplementary material for: Subcellular RNA profiling links splicing and nuclear DICER1 to alternative cleavage and polyadenylation
Source: Genome Res. 2016 Jan;26(1):24–35. doi: 10.1101/gr.193995.115 (PMC4691748; doi:10.1101/gr.193995.115)
Supplement: Supplemental Material [file supp_gr.193995.115_Supplemental_Figures.pdf]

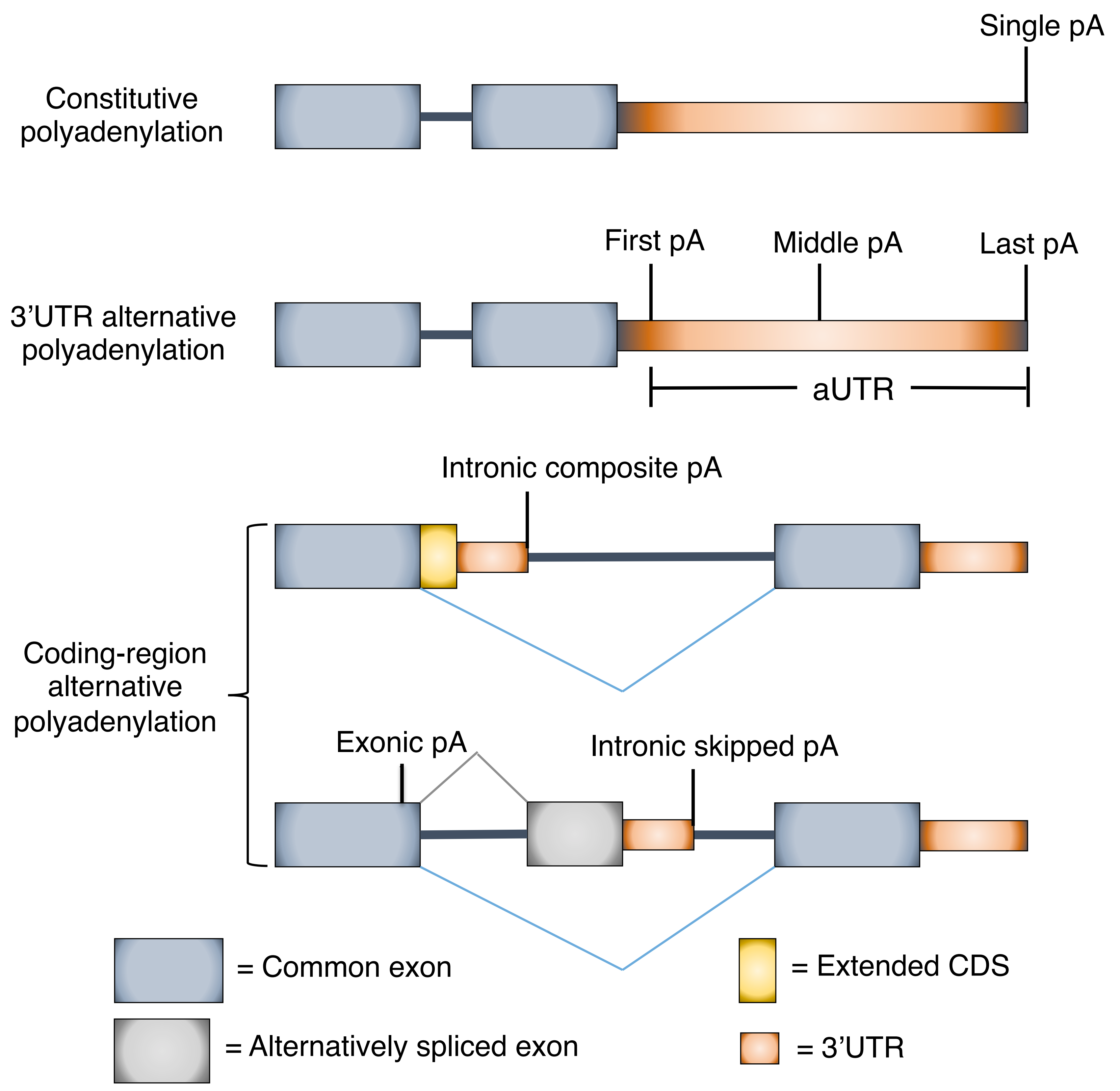
Single pA
Constitutive polyadenylation
First pA
Middle pA
Last pA
3'UTR alternative polyadenylation
aUTR
Intronic composite pA
Coding-region alternative polyadenylation
Exonic pA
Intronic skipped pA
= Common exon
= Extended CDS
= Alternatively spliced exon
= 3'UTR

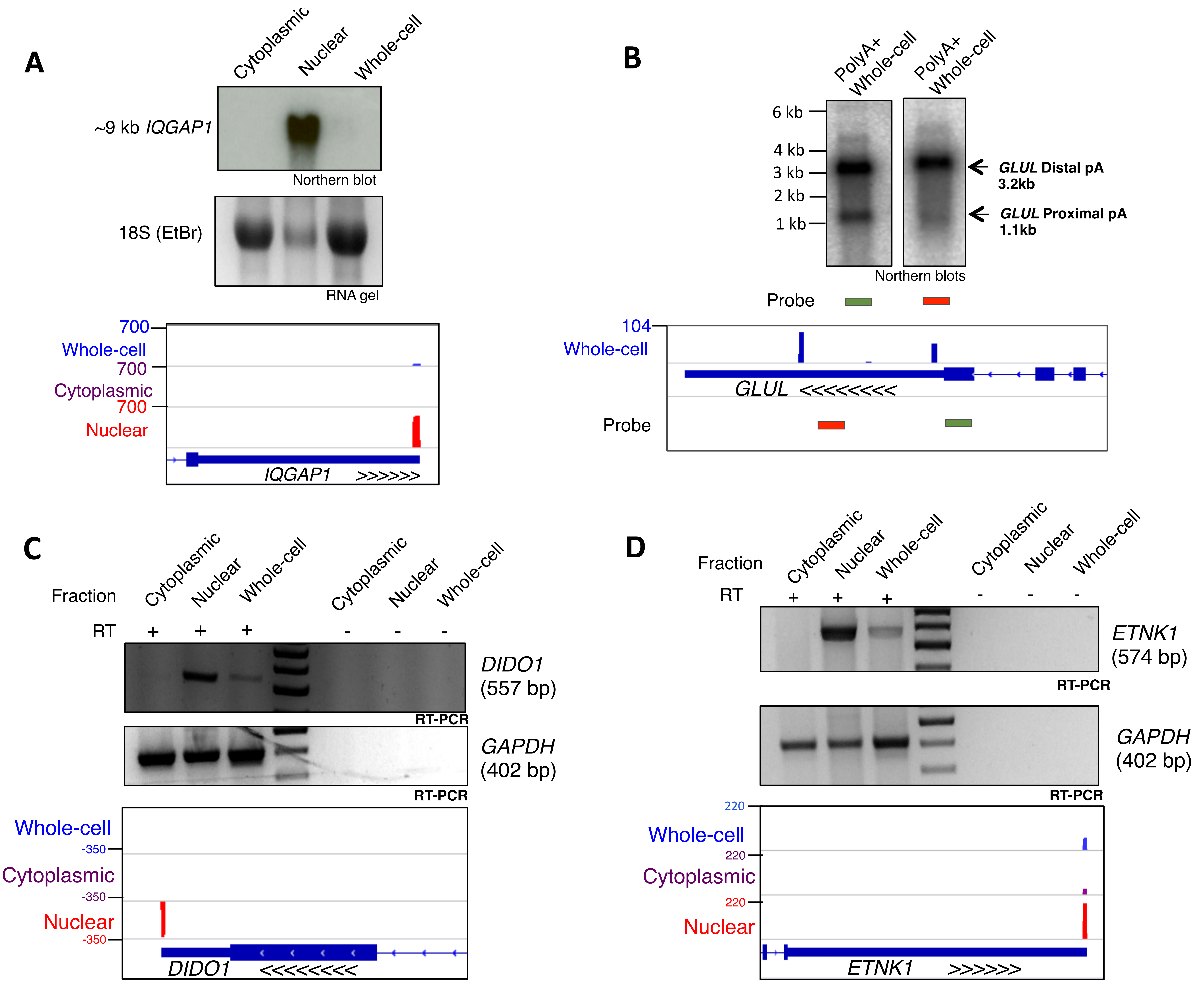
A
Cytoplasmic
Nuclear
Whole-cell
~9 kb IQGAP1
Northern blot
18S (EtBr)
RNA gel
700
Whole-cell
700
Cytoplasmic
700
Nuclear
IQGAP1 >>>>>>
B
PolyA+ Whole-cell
PolyA+ Whole-cell
6 kb
4 kb
3 kb
2 kb
1 kb
GLUL Distal pA 3.2kb
GLUL Proximal pA 1.1kb
Northern blots
Probe
104
Whole-cell
GLUL <<<<<<<<
Probe
C
Fraction
Cytoplasmic
Nuclear
Whole-cell
Cytoplasmic
Nuclear
Whole-cell
RT + + + - - -
DIDO1 (557 bp)
RT-PCR
GAPDH (402 bp)
RT-PCR
Whole-cell
-350
Cytoplasmic
-350
Nuclear
-350
DIDO1 <<<<<<<<
D
Fraction
Cytoplasmic
Nuclear
Whole-cell
Cytoplasmic
Nuclear
Whole-cell
RT + + + - - -
ETNK1 (574 bp)
RT-PCR
GAPDH (402 bp)
RT-PCR
220
Whole-cell
220
Cytoplasmic
220
Nuclear
ETNK1 >>>>>>

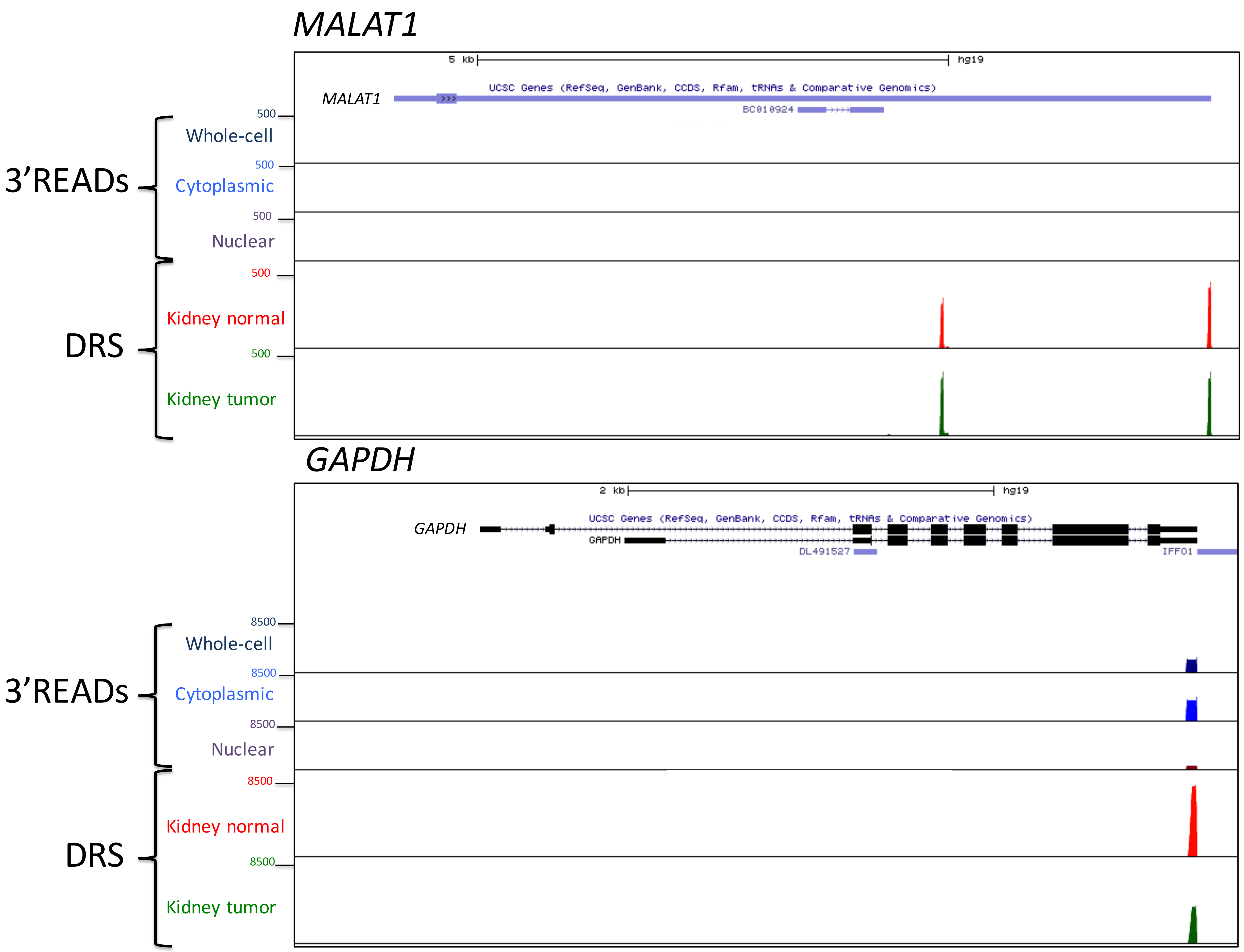
MALAT1
5 kb
hg19
UCSC Genes (RefSeq, GenBank, CCDS, Rfam, tRNAs & Comparative Genomics)
MALAT1
BC010924
500
Whole-cell
500
Cytoplasmic
500
Nuclear
3'READs
500
Kidney normal
500
Kidney tumor
DRS
GAPDH
2 kb
hg19
UCSC Genes (RefSeq, GenBank, CCDS, Rfam, tRNAs & Comparative Genomics)
GAPDH
GAPDH
DL491527
IFFO1
8500
Whole-cell
8500
Cytoplasmic
8500
Nuclear
3'READs
8500
Kidney normal
8500
Kidney tumor
DRS

Top 3 hits MCF7 (Shortening)

	Motif	Logo	E-value	Hits on Target database Ray et al. 2013
1.	CVGCCTCC	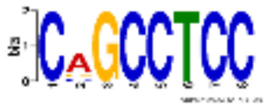	2.6e-033	-
2.	GGGAYTAC	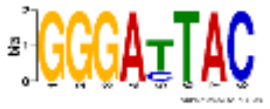	2.1e-027	NONO
3.	CAGGCTGG	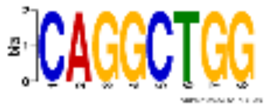	1.5e-025	-

Top 3 hits HEPG2 (Shortening)

	Motif	Logo	E-value	Hits on Target database Ray et al. 2013
1.	GGGATTAC	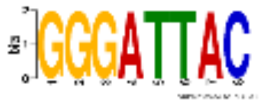	3.3e-017	-
2.	GAGGCKGA	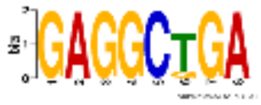	1.8e-016	-
3.	GTGGCTCA	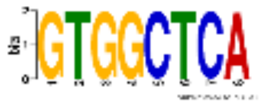	4.3e-016	-

Top 3 hits K562 (Shortening)

	Motif	Logo	E-value	Hits on Target database Ray et al. 2013
1.	AATAAA	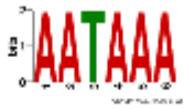	3.3e-019	KHDRBS1/2/3, ZFP36, PABPC1/4, RBM46
2.	GCWCACRC	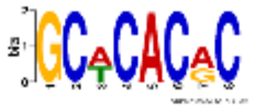	1.1e-012	hnRNPLL, RBMY1A1
3.	TAHAAATA	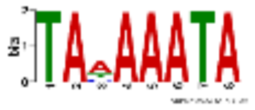	2.8e-012	-

Top 3 hits HeLaS3 (Shortening)

	Motif	Logo	E-value	Hits on Target database Ray et al. 2013
1.	AWATAMAA	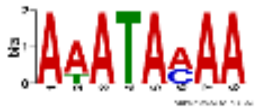	7.9e-014	-
2.	TTTTTAAW	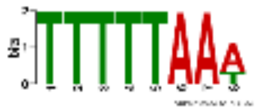	3.5e-013	hnRNPC, hnRNPCL1, RALY, CPEB4, TIA1, ZFP36
3.	GAGGCKGA	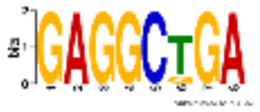	2.4e-009	-

Top 3 hits IMR90 (Shortening)

	Motif	Logo	E-value	Hits on Target database Ray et al. 2013
1.	DAAATAMA	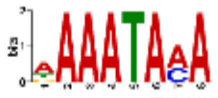	3.8e-019	ZFP36, PABPC1/4
2.	CWGCCTCC	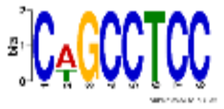	2.4e-013	-
3.	ATCCCAGC	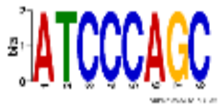	2.5e-012	-

Top 3 hits HUVEC (Shortening)

	Motif	Logo	E-value	Hits on Target database Ray et al. 2013
1.	AAAAWAAA	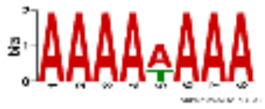	1.0e-012	ZFP36, PABPC1/3/4, SART3, PABPN1,KHDRBS2/3
2.	CCAGCTAC	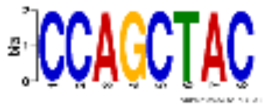	9.4e-012	-
3.	GATTACAK	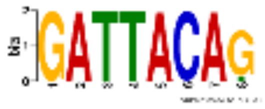	7.5e-010	-

Top 3 hits SKNSH (Shortening)

	Motif	Logo	E-value	Hits on Target database Ray et al. 2013
1.	AGGCYGAG	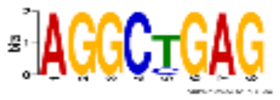	2.0e-022	-
2.	CTCACTGC	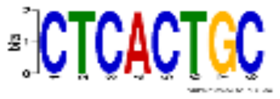	1.7e-020	-
3.	CRCCTGTA	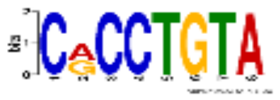	7.8e-020	Pum2 (*Mus Musculus*)

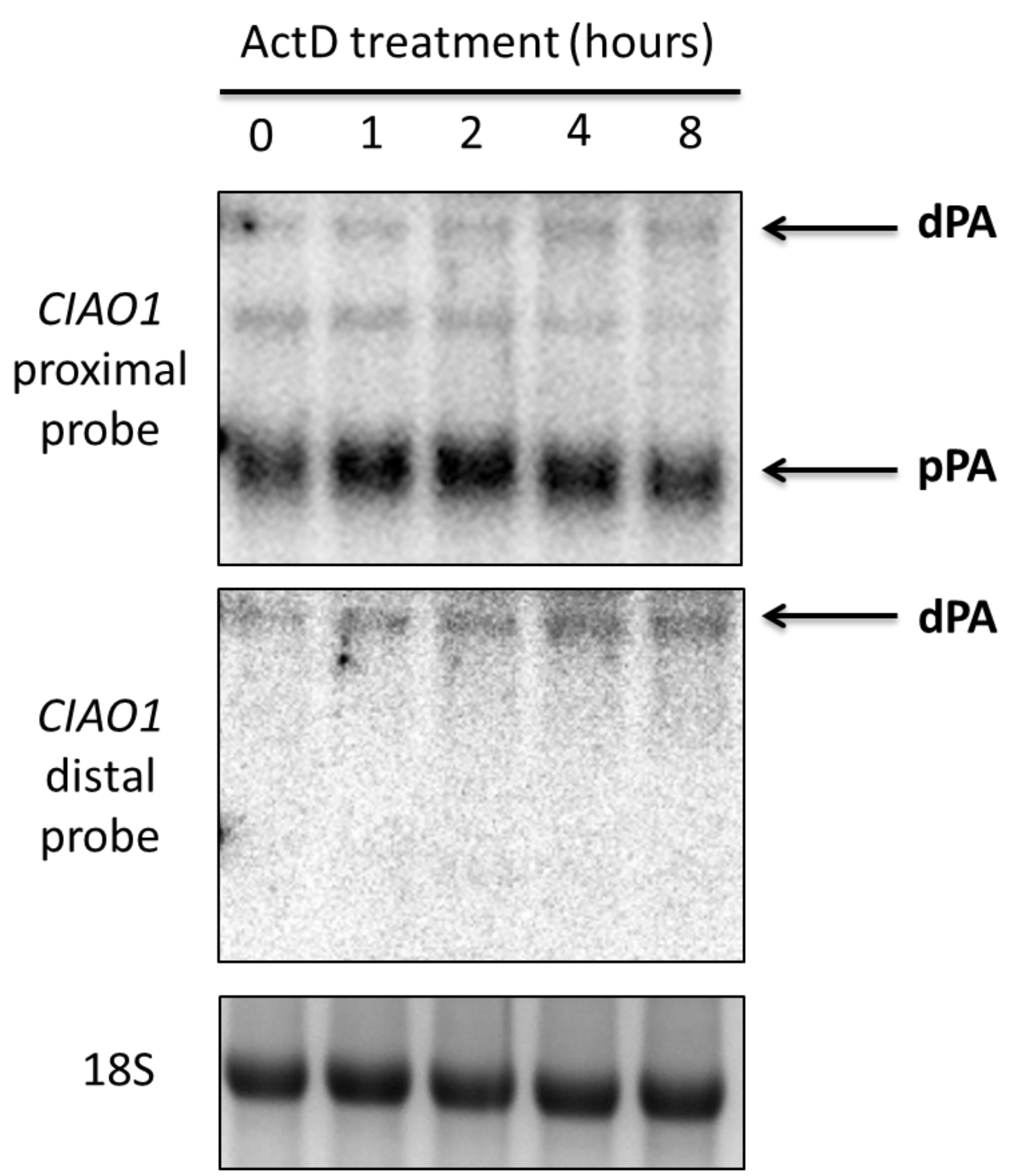
ActD treatment (hours)
0
1
2
4
8
CIAO1 proximal probe
CIAO1 distal probe
18S
dPA
pPA
dPA

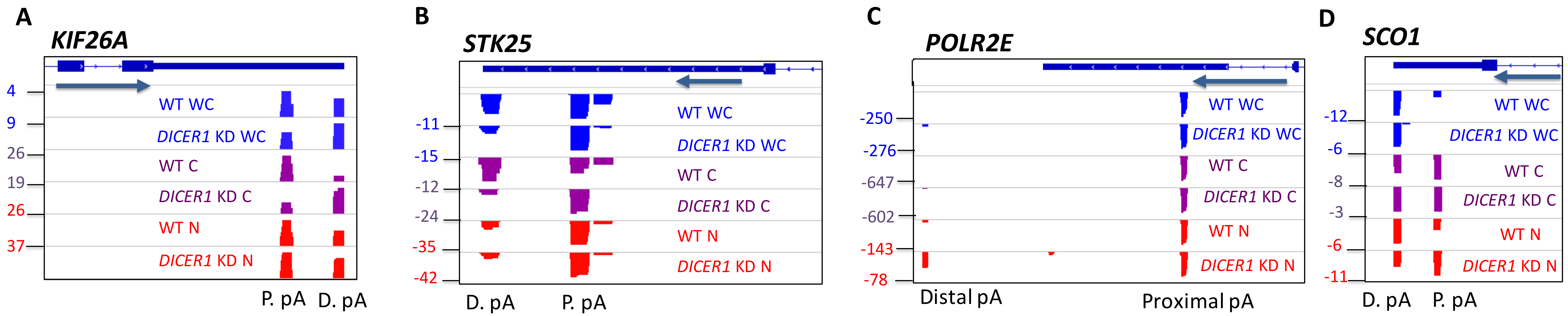
A
KIF26A
4
9
26
19
26
37
WT WC
DICER1 KD WC
WT C
DICER1 KD C
WT N
DICER1 KD N
P. pA
D. pA
B
STK25
-11
-15
-12
-24
-35
-42
WT WC
DICER1 KD WC
WT C
DICER1 KD C
WT N
DICER1 KD N
D. pA
P. pA
C
POLR2E
-250
-276
-647
-602
-143
-78
WT WC
DICER1 KD WC
WT C
DICER1 KD C
WT N
DICER1 KD N
Distal pA
Proximal pA
D
SCO1
-12
-6
-8
-3
-6
-11
WT WC
DICER1 KD WC
WT C
DICER1 KD C
WT N
DICER1 KD N
D. pA
P. pA

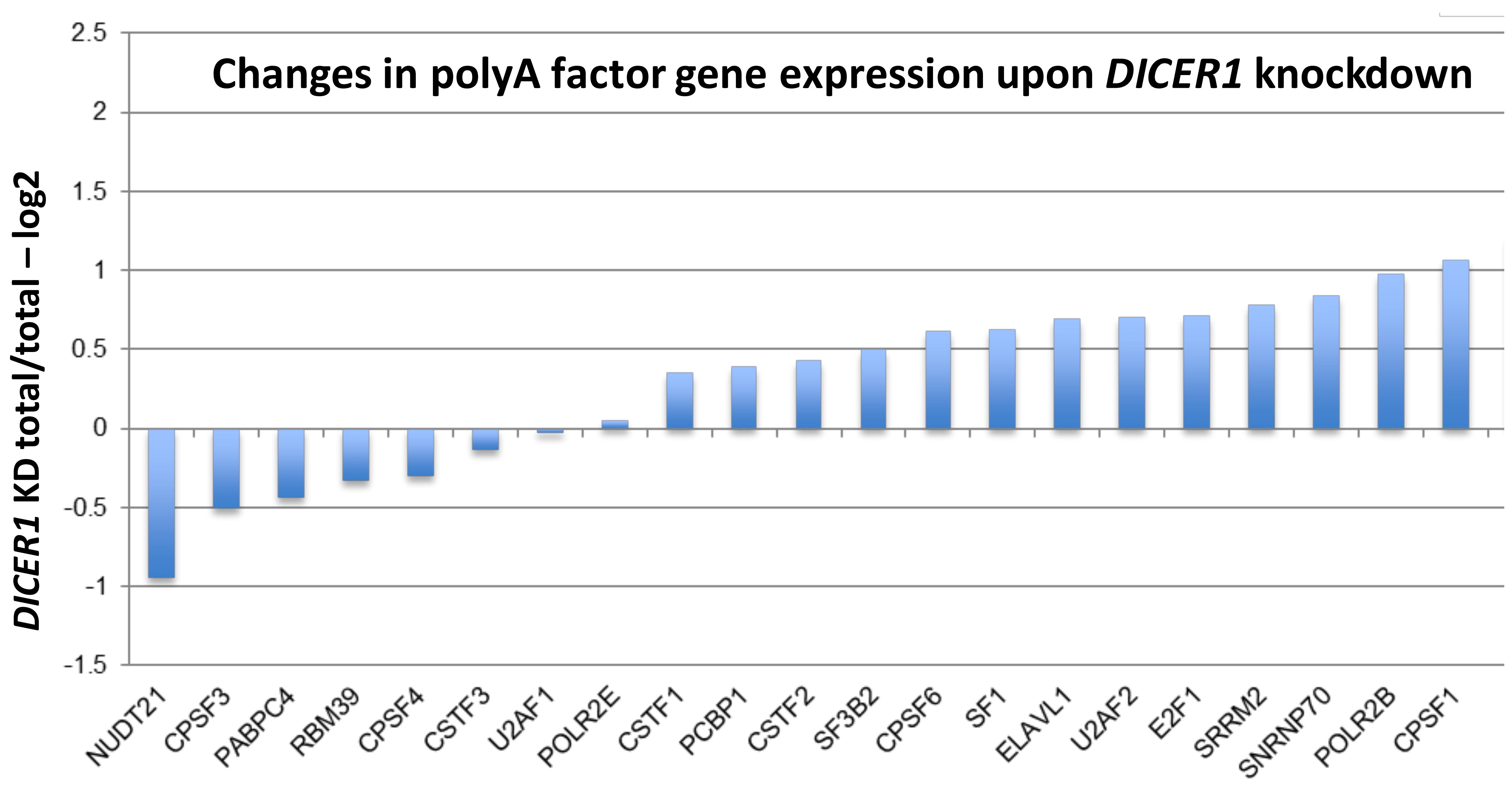
Changes in polyA factor gene expression upon DICER1 knockdown
DICER1 KD total/total – log2
2.5
2
1.5
1
0.5
0
-0.5
-1
-1.5
NUDT21
CPSF3
PABPC4
RBM39
CPSF4
CSTF3
U2AF1
POLR2E
CSTF1
PCBP1
CSTF2
SF3B2
CPSF6
SF1
ELAVL1
U2AF2
E2F1
SRRM2
SNRNP70
POLR2B
CPSF1

Neve_Figure S8

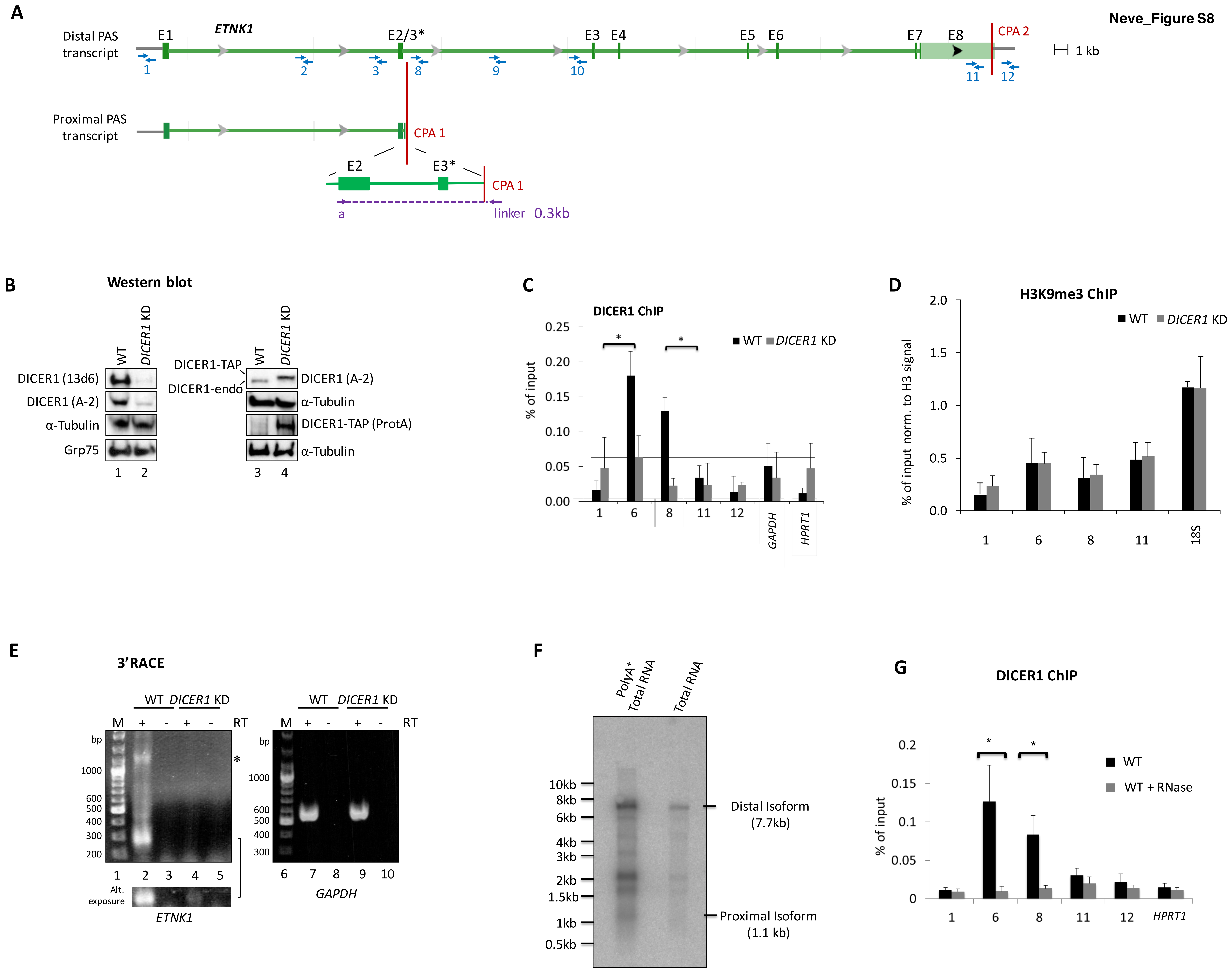

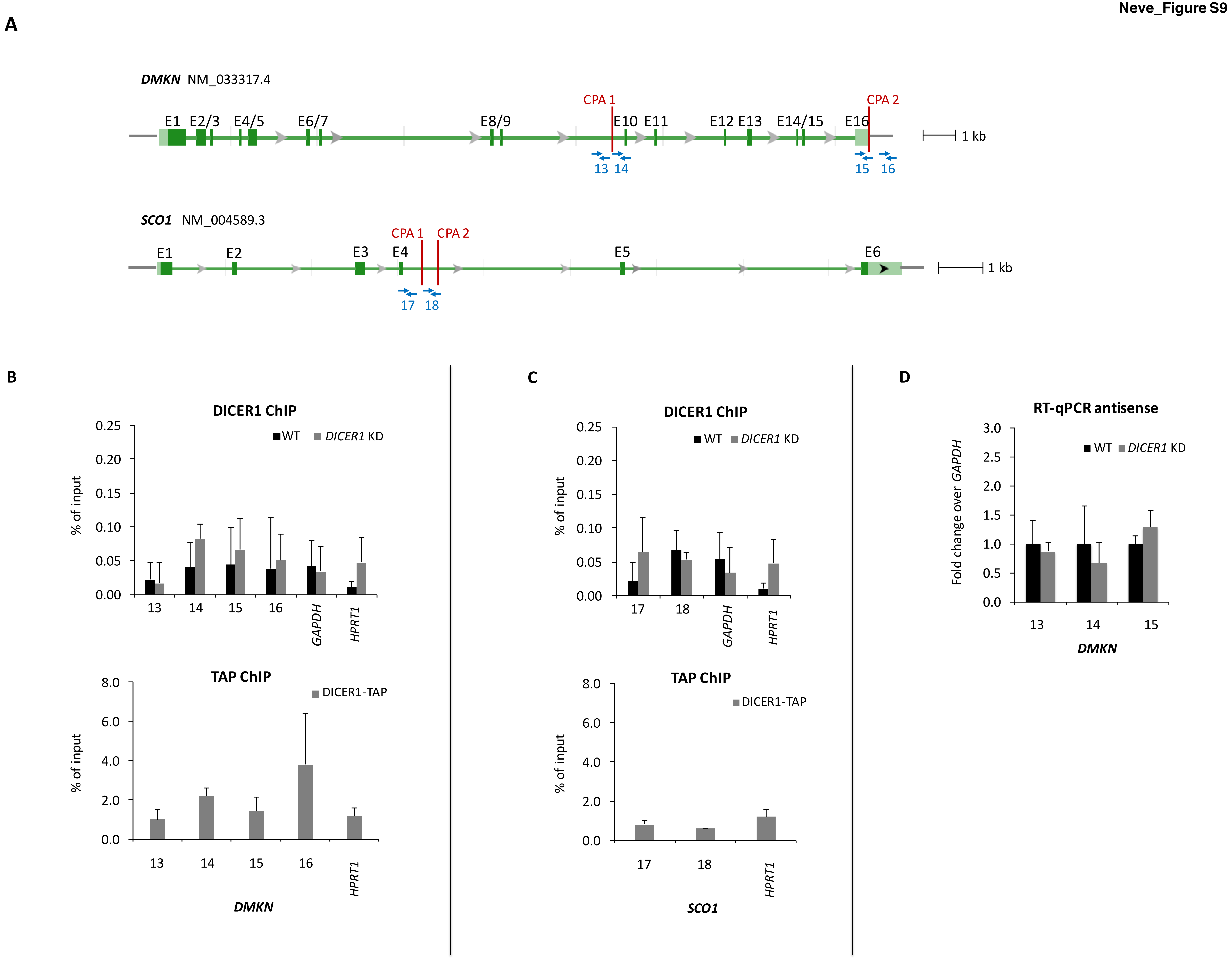
A
DMKN NM_033317.4
CPA 1
CPA 2
E1 E2/3 E4/5 E6/7 E8/9 E10 E11 E12 E13 E14/15 E16
1 kb
13 14 15 16
SCO1 NM_004589.3
CPA 1 CPA 2
E1 E2 E3 E4 E5 E6
1 kb
17 18
B
DICER1 ChIP
WT DICER1 KD
% of input
0.25 0.20 0.15 0.10 0.05 0.00
13 14 15 16 GAPDH HPRT1
TAP ChIP
DICER1-TAP
% of input
8.0 6.0 4.0 2.0 0.0
13 14 15 16 HPRT1
DMKN
C
DICER1 ChIP
WT DICER1 KD
% of input
0.25 0.20 0.15 0.10 0.05 0.00
17 18 GAPDH HPRT1
TAP ChIP
DICER1-TAP
% of input
8.0 6.0 4.0 2.0 0.0
17 18 HPRT1
SCO1
D
RT-qPCR antisense
WT DICER1 KD
Fold change over GAPDH
3.0 2.5 2.0 1.5 1.0 0.5 0.0
13 14 15
DMKN

Western Blot

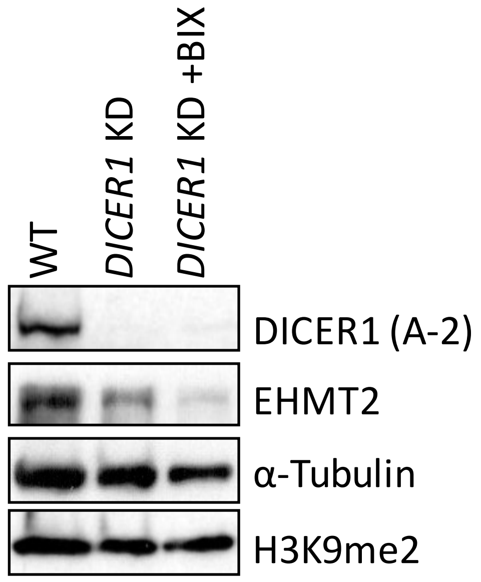

A

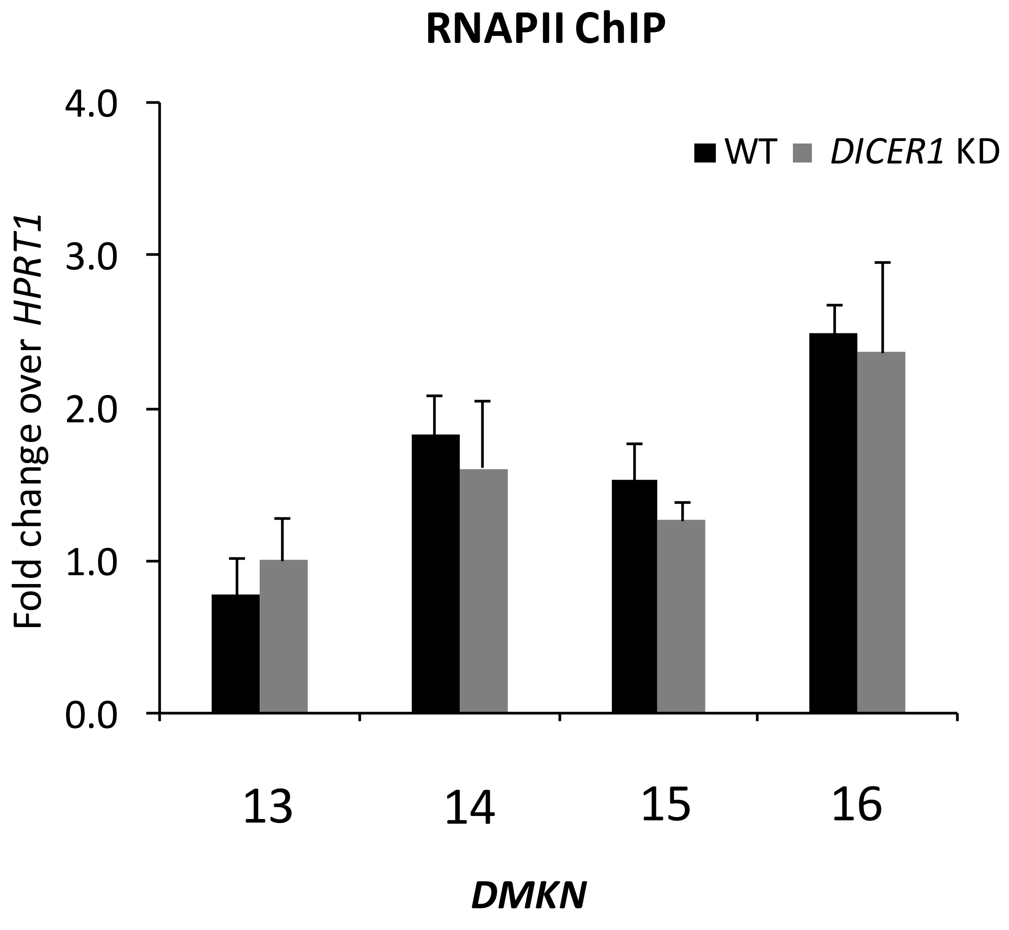
RNAPII ChIP
Fold change over HPRT1
4.0
3.0
2.0
1.0
0.0
WT
DICER1 KD
13
14
15
16
DMKN

B

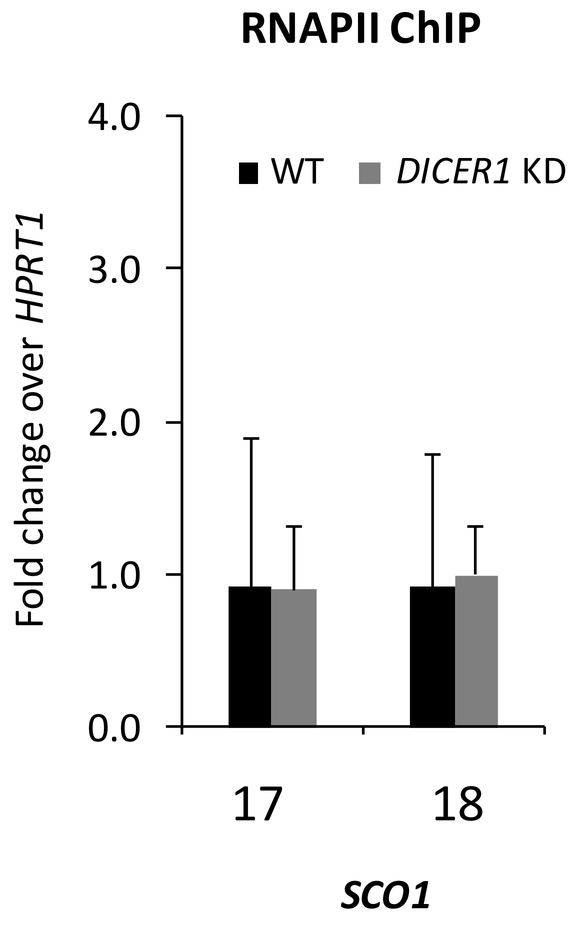
RNAPII ChIP
Fold change over HPRT1
4.0
3.0
2.0
1.0
0.0
WT
DICER1 KD
17
18
SCO1
