# Supplementary material for: Subcellular RNA profiling links splicing and nuclear DICER1 to alternative cleavage and polyadenylation
Source: Genome Res. 2016 Jan;26(1):24–35. doi: 10.1101/gr.193995.115 (PMC4691748; doi:10.1101/gr.193995.115)
Supplement: Supplemental Material [file supp_gr.193995.115_Supplemental_Information.docx]

**SUPPLEMENTAL INFORMATION**

**Figure Legends**

**Figure S1: Schematic Representation of Types of Polyadenylation.**

Constitutive polyadenylation occurs when there is a single cleavage and polyadenylation (CPA) site for a gene, resulting in all transcript isoforms having an identical 3’-untranslated region (3’UTR). 3’UTR alternative polyadenylation (APA) occurs when there are multiple CPA sites contained within the 3’UTR of a single gene, with the region contained between the CPA sites known as the alternative UTR (aUTR). 3’UTR-APA can therefore produce transcript isoforms that contain the same coding-region, and thus produce identical proteins, but differ in their 3’UTR length. The CPA sites in the 3’UTR are designated first, middle and last, depending on their positioning relative to other CPA sites. Coding-region APA (CR-APA) occurs when CPA sites are located upstream of the terminal 3’UTR, and thus result in proteins with distinct C-terminal coding regions. These CPA sites can be located within the coding region itself, exonic pA, within introns when a splice site is not used, intronic-constitutive pA, or in an intronic regions which are coupled with skipped terminal exons, intronic-skipped pA.

- **Figure S2: Confirmation of mRNA distribution as measured by 3’READS.**

(A) Upper panel shows a northern blot confirming the subcellular distribution of *IQGAP1* mRNA as measured by 3’READS (lower panel). The middle panel acts as a loading control showing the 18S ribosomal RNAs on a denaturing agarose gel, where 15 ug of RNA from each subcellular fraction was loaded.

(B) Northern blot of whole cell polyA+ HEK293 RNA corroborating the 2 UTR-APA isoforms of *GLUL* identified by 3’READS and their respective abundances. The 3’READS trace for whole-cell HEK293 RNA is shown in the lower panel.

(C) and (D) Reverse-transcription PCR confirming the subcellular distribution of *DIDO1* and *ETNK1* genes respectively, as measured by 3’READS. The distribution of read numbers as measured by 3’READS is shown in the lower panels. RT-PCRs of *GAPDH* were used as loading controls.

**Figure S3: Quality control for amplification of solely polyadenylated transcripts.**

Screenshots of the UCSC genome browser of the *MALAT1* and *GAPDH* genes compares the 3’READS method to direct RNA sequencing (DRS). *MALAT1* contains a well-characterised genomically-encoded oligo-adenylated tail. This transcript is only mapped by the DRS method. Conversely the lower pane shows *GAPDH*, which is a truly polyadenylated transcript, is picked up by both 3’READS and DRS. The strong preference of the 3’READS method for polyadenylated over oligoadenylated mRNAs is underpinned by the low numbers of reads that can be mapped to the oligoadenylated *MALAT1* transcript (Wilusz and Spector 2010). This demonstrates that 3’READS is a stringent method of accurately mapping solely CPA sites.

**Figure S4: Motif analysis of aUTR regions in genes that show overrepresentation of shorter APA isoforms in the cytoplasm.**

The aUTRs of all genes that showed overrepresentation of the shorter APA isoform in the cytoplasm in each individual cell line were extracted and subjected to motif analysis using the DREME tool, with a scrambled reference file. The top 3 motifs for each cell line are shown as well as corresponding RNA-binding proteins that were found to bind these motifs (Ray et al. 2013).

**Figure S5**: **Stability experiment *CIAO1.***

HEK293 cells were exposed to Actinomycin D (ActD) for the times indicated. Total RNA was isolated at each time point and subjected to northern blotting with radioactive probes that detect both proximal and distal APA isoforms (proximal probe) and distal APA isoforms only (distal probe) respectively.

**Figure S6**: **Screenshots of UTR-APA upon DICER1 depletion in HEK293s.**

(A) Screenshot of *KIF26A*, showing 3’UTR lengthening in the *DICER1* KD cells compared to cytoplasmic fractions, with read numbers on the left.

(B, C, D) As in Panel A, *STK25* shows 3’UTR shortening. *POLR2E* and *SCO1* show lengthening and shortening of 3’UTRs respectively, when comparing the nuclear fractions of the *DICER1* KD cells to the control cells. The positions of these genes on the scatter plots are highlighted in Figures 5 B, C and D.

**Figure S7**: **Changes in gene expression upon *DICER1* knockdown of known effectors of CPA site choice.**

The gene expression of genes that have previously been linked to affecting alternative polyadenylation was compared in control and *DICER1* KD whole cells fractions. Genes were only included if at least one of the samples had greater than 10 reads per million.

**Figure S8: Validation of knockdown efficacy and sequencing results at the *ETNK1* locus.**

(A) Schematic showing positions of probes for 3’RACE (a, linker) and ChIP amplicons (1-12) at the human ethanolamine kinase 1 (*ETNK1*) locus around proximal and distal CPA sites 1 and 2 (CPA 1 and 2). E3*: alternative exon 3.

(B) Western Blot showing depletion of endogenous DICER1 (lanes 1, 2) and reconstitution with recombinant, TAP-tagged DICER1 in *DICER1* KD HEK293 cells (lanes 3, 4). Grp75: 75 KDa glucose-regulated protein.

(C, D) ChIP analysis of DICER1 (C) and histone 3 lysine 9 trimethylation (H3K9me3) (D) surrounding the *ETNK1* CPA sites (6, 8: intronic CPA 1; 11, 12: canonical CPA 2). Values from non-induced, *DICER1* KD HEK293 cells are shown as % of input. Mean values and standard deviations were calculated from at least three biological replicates. *p* < 0.05 (*) by two-tailed Student's *t* test. HPRT1: hypoxanthine phosphoribosyltransferase 1; 18S: 18S ribosomal RNA.

(E) Rapid amplification of 3’ ends (3’RACE) showing transcript levels of *ETNK1* and *GAPDH* in wild type and *DICER1* KD HEK293 cells. Asterisk: unspecific band.

(F) Northern blot of *ETNK1* using a probe for exon 1, as shown in Panel A. The RNA probed was 3 ug of polyA+ RNA isolated from HEK293 whole-cell RNA. 3ug of the input used for the polyA+ purification was used as a reference. The expected sizes of the proximal and distal APA isoforms, as shown in panel A are indicated.

(G) ChIP analysis of DICER1 performed as described above but with an additional RNase treatment (mix of 7.5 U of RNase A and 300 U of RNase T1) for 30 minutes at RT prior to immune precipitation.

**Figure S9: DICER1 occupancy at proximal and distal CPA sites of the *DMKN* and *SCO1* loci.**

(A) Schematic showing positions of ChIP amplicons (13-18) at the Dermokine (*DMKN*) and Cytochrome C Oxidase Assembly Protein (*SCO1*) loci around proximal and CPA sites 1 and 2 (CPA 1 and 2).

(B, C) ChIP analysis of endogenous and recombinant, TAP-tagged DICER1 around CPA 1 and 2 shown as % of input. Mean values and standard deviations were calculated from at least three biological replicates. *p* < 0.05 (*) by two-tailed Student's *t* test. *GAPDH*: glyceraldehyde-3-phosphate dehydrogenase, *HPRT1*: hypoxanthine phosphoribosyltransferase 1.

(D) Antisense RNA levels around *DMKN* at CPA 1 and 2. Primer pairs specific for antisense transcripts were used to determine relative changes in antisense RNA levels upon *DICER1* KD by RT-qPCR.

**Figure S10: BIX treatment impairs EHMT2 expression and cell proliferation.**

Western blot showing EHMT2 expression upon DICER1 depletion and BIX treatment. Wild type and *DICER1* KD HEK293 cells were incubated EHMT2-specific small molecule inhibitor BIX (BIX-01294, 5 μM, 72 hrs).

**Figure S11: ChIP analysis of RNAPII around *DMKN* and *SCO1.***

(A, B) RNA polymerase II (RNAPII) occupancy at human *DMKN* (A) and *SCO1* (B) CPA sites. % of input values from non-induced and *DICER1* KD HEK293 cells are shown as fold change over HPRT1 signals. Probe positions are shown in Figure S9A.

**Detailed Methods:**

**Subcellular Fractionation**

Around 6 x 10^7^ cells were trypsinized, washed 2 times in ice cold PBS and the cell pellet was then resuspended with slow pipetting in 1 ml of Lysis Buffer B (10 mM Tris-HCl (pH 8-8.4) 0.14 M NaCl, 1.5 mM MgCl_2_, 0.5 % NP-40). 100 μl of lysate was removed as the whole-cell RNA fraction. The nuclei were then spun down at 1000g for 4 minutes. The supernatant was removed representing the cytoplasmic fraction and spun at 11,000g for 1 minute to remove remaining nuclei. The nuclei pellet was resuspended in 1 ml of Lysis Buffer B and 1/10^th^ volume (100 μl) of the Detergent Stock Solution (3.3 % (w/v) sodium deoxycholate, 6.6 % (v/v) Tween 40) was added under slow vortexing. The stripped nuclei were then spun down at 1000g for 4 minutes at 4°C. The nuclei pellet was then washed once more in Lysis Buffer B and spun down at 1000g for 4 minutes at 4°C. The nuclei pellet was then resuspended in 1 ml of TRIzol using a 21-gauge syringe. TRIzol was also used to extract RNA from the cytoplasmic and whole cell fractions as per manufacturer’s instructions. The RNA pellets were then DNase I-treated (Roche) as per manufacturer’s instructions. RNA was phenol-chloroform extracted and precipitated with ethanol and concentrations determined using a NanoDrop ND1000.

**3’READS Protocol**

The 3’READS protocol was originally described in (Hoque et al. 2013). Briefly, 25-30 μg of RNA was subjected to 1 round of poly(A) selection using the Poly(A)Purist^TM^ MAG kit (Ambion) according to the manufacturer’s protocol, followed by fragmentation using Ambion’s RNA fragmentation kit at 70°C for 5 min. Poly(A)-containing RNA fragments were isolated using the CU5T45 oligo (a chimeric oligo containing 5 Us and 45 Ts, Sigma) which were bound to the MyOne streptavidin C1 beads (Invitrogen) through biotin at its 5’ end. Binding of RNA with CU5T45 oligo-coated beads was carried out at room temperature for 1 hr in 1x binding buffer (10 mM Tris-HCl pH 7.5, 150 mM NaCl, 1 mM EDTA), followed by washing with a low salt buffer (10 mM Tris-HCl pH 7.5, 1 mM NaCl, 1 mM EDTA, 10% formamide). RNA bound to the CU5T45 oligo was digested with RNase H (5U in 50 µl reaction volume) at 37°C for 1 hr, which also eluted RNA from the beads. Eluted RNA fragments were purified by phenol:chloroform extraction and ethanol precipitation, followed by phosphorylation of the 5' end with T4 kinase (NEB). Phosphorylated RNA was then purified by the RNeasy kit (Qiagen) and was sequentially ligated to a 5’-adenylated 3’-adapter (5’-rApp/NNNNGATCGTCGGACTGTAGAACTCTGAAC/3ddC) with the truncated T4 RNA ligase II (Bioo Scientific) and to a 5’ adapter (5’- GUUCAGAGUUCUACAGUCCGACGAUC) with T4 RNA ligase I (NEB). The resultant RNA was reverse-transcribed to cDNA with Superscript III (Invitrogen), and the cDNA was amplified by 12 cycles of PCR with Phusion high fidelity polymerase (NEB). cDNA libraries were sequenced on an Illumina HiSeq 2500.

**Analysis of 3’READS data**

Raw reads were mapped to the human genome (hg19) with bowtie2 using the option “-M 8 --local”. This mode allows the ends of the sequence not to be part of the alignment (soft clipped). This is useful for reads containing sequence corresponding to the poly(A) tail which are not present in the genomic sequence. Reads that were shorter than 15 nt, were non-uniquely mapped to genome (MAPQ<10), or contained more than 2 mismatches in alignment were discarded. Reads containing ≥ 2 extra (not aligned with the genome) Ts at the 5’end were selected as polyA site supporting (PASS) reads. Generally an individual CPA was assigned if it is identified by >5% of all reads mapped to a particular gene.

CPA sites located within 24 nt as mapped by PASS reads from all samples were clustered. The site with the most PASS reads was selected to represent the CPA site cluster. All the reads in a cluster were used to calculate isoform abundance. CPA sites were mapped to RefSeq transcripts. CPA sites located downstream of RefSeq transcripts were linked to the transcript by cDNA, EST and directional paired-end RNA-seq data from the ENCODE project. An extended region is covered by cDNA/EST sequences or RNA-seq reads without a gap greater than 40 nt. We also required that the 3’UTR extension do not exceed transcriptional start site or 3’splice site of any other gene on the same strand. The relative abundance of a transcript isoform in a sample was defined as the proportion of PASS reads supporting this isoform in all the PASS reads mapped to the gene. To remove data noise, CPA sites with relative abundance <5% in all of the samples were discarded.

To cross-compare global mRNA isoforms abundances as described in Figure 1B, CPA isoforms were ranked by mean log2 RPM values and the top 50% of the highly expressed CPA isoforms were selected. The log2 RPM values were then mean-centred and the top 5,000 expression-variable CPA isoforms were further selected and used for hierarchical clustering.

**Significance Analysis of Alternative Polyadenylation (SAAP)**

SAAP analysis was performed as in (Li et al. 2015). Briefly, C/N index was calculated based on (C-N)/(C+N), in which “C” and “N” represent the read per million total read (RPM) values of the CPA site for cytoplasmic and nuclear fractions. To study the difference of C/N index between APA isoforms in 3’UTR of genes, we first bootstrapped total PASS read number to 1 million. This allows us to control the impact of sequencing depth. For each gene, 2 most expressed 3’UTR CPA sites were selected for study. The observed delta-C/N indexes were calculated between the two CPA sites. To get the expected delta-C/N index, we bootstrapped reads for the two CPA sites in cytoplasmic and nuclear fractions by assuming that the RPM ratio between cytoplasmic and nuclear fractions follows the averaged ratio for the two CPA sites. We bootstrapped the reads and calculated expected delta-C/N index for 100 times for each gene. Based on the 100 expected values, we converted all observed and expected delta-C/N indexes to Z scores. For the whole data set, we select genes with significant C/N index difference between CPA sites by choosing observed absolute Z scores greater than a cutoff (Z_cut). The False Discovery Rate (FDR) was estimated by calculating the fraction of the average number of gene with |expected Z scores|> Z_cut among the number of gene with |observed Z scores|> Z_cut. The FDR were controlled at <10% for this study. The whole SAAP method was performed 20 times for each cell type.

**Reverse Transcription PCR and RNA Gel Analysis**

1 μg of DNase-treated RNA was reverse transcribed into cDNA using RevertAid reverse transcriptase (Fermentas) as per manufacturer’s instructions with an oligo(dT) primer. For northern blots, purified RNA was resolved on 1% formaldehyde-agarose gels. ^32^P-labelled probes were synthesised using asymmetric PCR from gel purified RT-PCR products and quantitation was performed on a phosphoimager.

**Western Blot**

Non-induced and Doxycycline-induced HEK293 cell line 2.B were collected from 75 cm^2^ flasks and lysed in 4 x SDS loading buffer. Lysates were sonicated, boiled, separated on pre-cast SDS polyacrylamide gel. The membrane was blocked in 5% milk and incubated with specific primary antibodies at 4°C overnight. After washing, membranes were incubated with horseradish peroxidase (HRP)-conjugated secondary antibodies for 1.5 hours at RT, washed and signals were detected using enhanced chemiluminescence (ECL) kit (Perkin Elmer).

**Chromatin immunoprecipitation**

Non-induced, Doxycycline-induced (3 µg/ml, 7 days) HEK293 cells (2.B and 1.3 cells) were collected from 175 cm^2^ flasks. Treatment with BIX 01294 trihydrochloride hydrate (BIX-01294, Sigma Aldrich) was 5 μM for 72 hours prior to ChIP. 37 % formaldehyde was added directly to the tissue culture medium at final concentration of 1% and then incubated for 10 min at 37 °C. Formaldehyde was inactivated by adding glycine to a final concentration of 0.125 M. Cells were washed twice with 5 ml ice-cold PBS containing protease and phosphatase inhibitors (Sigma Aldrich) and then scraped into 15ml tubes. Samples were centrifuged for 5 min at 1600 r.p.m. and 4 °C. Cells were gently resuspended in 500 μl of cell lysis buffer (5 mM PIPES, pH 8.0, 85 mM KCl, 0.5% Nonidet P-40, 1 mM PMSF, 1 μg/ml pepstatin A, 1 μg/ml leupeptin and 1 x protease inhibitor cocktail) and incubated on ice for 10 min. Nuclei were collected by centrifugation for 5 min at 3000 r.p.m. and 4 °C and resuspended in 400 μl ice-cold nuclear lysis buffer (1% SDS, 10 mM EDTA, 50 mM Tris-HCl, pH 8.0, 0.5 mM PMSF, 0.8 μg/ml pepstatin A, 1 μg/ml leupeptin and 1 x protease inhibitor cocktail) and then incubated on ice for 10 min. Samples were sonicated to an average length of 300–500 bp, kept on ice (15 s sonication and 15 s rest) and spun for 10 min at 13,000 r.p.m. and 4 °C to remove cell debris. The supernatant was diluted by adding immunoprecipitation dilution buffer (0.01% SDS, 1.1% Triton X-100, 1.2 mM EDTA, 16.7 mM Tris-HCl, pH 8.1, 167 mM NaCl, 0.5 mM PMSF, 0.8 μg/ml pepstatin A, 1 μg/ml leupeptin and 1 x protease inhibitor cocktail (Sigma Aldrich)) and aliquoted into various immunoprecipitation samples. Specific antibodies (5 μg/100 μg chromatin) were added to samples and incubated overnight at 4 °C on a rotating wheel. Immune complexes were pulled down at 4 °C with 40 μl of protein A or G agarose beads pre-blocked with salmon sperm DNA (16-157, Millipore) or IgG Sepharose 6 fast flow beads (GE healthcare) and then washed extensively with buffers A–D: A, 0.1% SDS, 1% Triton X-100, 2 mM EDTA, 20 mM Tris-HCl, pH 8.0, and 150 mM NaCl; B, 0.1% SDS, 1% Triton X-100, 2 mM EDTA, 20 mM Tris-HCl, pH 8.0, and 500 mM NaCl; C, 0.25 M LiCl, 1% NP-40, 1% sodium deoxycholate, 1 mM EDTA and 10 mM Tris-HCl, pH 8.0; D. 10:1 TE, pH 8.0. Immune complexes were eluted with 500 μl immunoprecipitation elution buffer (1% SDS and 0.1 M NaHCO_3_) for 30 min on a rotating wheel. Reversal of cross-links was performed by adding 0.3 M NaCl, 3 μg/ml RNase A, 10 μl of 0.5M EDTA, 20 μl of 1 M Tris-HCl, pH 6.5 and 2 μl of 10 mg/ml proteinase K and incubating at 65 °C overnight. DNA was purified by Phenol/Chloroform extraction and recovered in 60 μl of ddH_2_O. Signals represent the average of at least three biological repeats expressed as a percentage of the input signal using real-time quantitative PCR (RT-qPCR) and comparative quantitation.

**Transient Transfection**

Non-induced and Doxycycline-induced HEK293 cell line 2.B was seeded on 9 cm^2^ dishes and incubated in OptiMEM medium (Invitrogen) for 2 hours prior to transfection. Transient transfection of recombinant, α-amanitin resistant RNA polymerase II (RNAPII) construct (WTR (pAT7Rpb1αAm^r^)), or slow elongating mutant thereof (C4 (pAT7Rpb1αAm^r^R749H)), (de la Mata et al. 2003) or α-amanitin sensitive mock plasmid pcDNA 5**/**FRT/TO (Invitrogen) was performed using Lipofectamine 2000 (Invitrogen) (1 µl/µg plasmid) according to manufacturer’s instructions. Plasmid integrity was confirmed by sequencing. Cells were co-transfected with enhanced green fluorescent protein (eGFP) expressing plasmid GW-pEGFP. Transfection efficacy was monitored by immunofluorescence microscopy. For selection of cells expressing α-amanitin resistant RNAPII constructs, transfected cells were cultured in presence of α-amanitin (2.5 µg/ml) for 48 hours. Total RNA was isolated and analyzed as described above.

**Antibodies**

Antibodies used: human anti-DICER1 (13d6, Abcam, ab14601); anti-DICER1 (A-2, Santa Cruz, sc-136981); anti-α-Tubulin (YL1/2, Abcam, ab6160); anti-RNAPII (N-20 X, Santa Cruz, sc-899 X); anti-Grp75 (JG1, Abcam, ab2799); anti-protein A (polyclonal, Abcam, ab7243); anti-EHMT2/G9a (Abcam, ab40542); anti-H3K9me2 (Abcam, ab1220); anti-H3K9me3 (GF12-H4, Merck Millipore, 05-1242); anti-TAP (IgG Sepharose 6 Fast Flow beads, GE Healthcare, 17-0969-01); anti-GAPDH (Sc-20356, Santa Cruz).

**Oligonucleotides**

|  |  |
| --- | --- |
| RT-PCR primers |  |
| *MALAT1* forward | CCTGCAAATTGTTAACAGAA |
| *MALAT1* reverse | TCAGCTTCCGCTAAGATGCTAGCTT |
| *GAPDH* forward | GGTGAAGGTCGGAGTCAACG |
| *GAPDH* reverse | TGGTTCACACCCATGACGAA |
| *DIDO1* forward | TGGGAAATTGCCTACGGAGC |
| *DIDO1* reverse | AGGCGAAACTGGACCAACAA |
| *ETNK1* forward | TGTTGAATTGGGAAATTGTGGCAT |
| *ETNK1* reverse | TTTTTTTTTTTTTGTAGTTAGAACTCAAC |
| Oligo(dT) RT primer | GCCCACGCGTCGACTAGTAC(T)_15_ |
| Primers for *CIAO1* northern probe northern*nNo*northerns |  |
| cUTR forward | TCCCAACTCGGATCCACAGC |
| cUTR reverse | CAAAGGCGGGGAAGGAAAGC |
| aUTR forward  a | GGCCACAAGCCCTGTATTCT |
| aUTR reverse | GTACAGCCCCACTGTCCTTC |
| intronic forward | GAGTCAGAGCAAATGGGGGT |
| intronic reverse | ACTGGCGTTTCTCAAGCAGA |
| Primers for *GLUL* northern probe |  |
| cUTR forward | ACTAAGCAAGCGGCACCAGT |
| cUTR reverse | ACTAGGAGGGGCTCAACAGC |
| aUTR forward  a | TTAATGGGGTGCACCTGTCC |
| aUTR reverse | AGGGCATTTAGTCCCAAGCC |
| Primers for *IQGAP1* northern probe |  |
| forward | TCAGAATGGGTCAAGGCACC |
| reverse | CGTGTAACATAGGTTGGCTGG |
| Primers for *ETNK1* northern probe |  |
| forward | CGTTCAGGATCAGGAGGAGC |
| reverse | GGGTTGCAGACATGCTTTGG |
| 3’RACE |  |
| Phased oligo d(T) | TTCGTATACCCGGGTACCAA(T)_15_N |
| *ETNK1* a-forward | AAACACCATGGAGGATGTAG |
| *GAPDH* b-forward | AAGGAAACTGTCTCGCATAG |
| linker | TTCGTATACCCGGGTACCAA |

| ChIP primers | |  |  |
| --- | --- | --- | --- |
|  |  |  |  |
| no. |  | Sequence (5' -3') |  |
| 1 | fwd | TGGGGTTATTTTAGAACGTG |  |
|  | rev | GAACTGCTAGTCTGGAAAGC |  |
| 2 | fwd | GGTGGATCAATCCCTTAGTC |  |
|  | rev | AATCTGCAGTTGCTTGAATC |  |
| 3 | fwd | TCCCATTCTCAGAGACTTTC |  |
|  | rev | CCTTCCTTGATCCAAAGTG |  |
| 4 | fwd | AAACACCATGGAGGATGTAG |  |
|  | rev | GATCCAGTGCTTCTCCTTG |  |
| 5 | fwd | TTATACAAGGAGAAGCACTGG |  |
|  | rev | ACAAAAACAAAAACCCTAGC |  |
| 6 | fwd | AATTGTCTTTGTTTGAGCAAC |  |
|  | rev | AAAACTAAGCAGAAGCCTTG |  |
| 7 | fwd | CAGAGGGTCAAGGCTTCTG |  |
|  | rev | AATAGGCAAGAACTCTGAAAAC |  |
| 8 | fwd | ACCAACTTCTTTTCATCACC |  |
|  | rev | CTCCACTCTGCTTCTCCTC |  |
| 9 | fwd | ACTGAAGGACATTTTGGTTG |  |
|  | rev | AAAATAGCACAGCCATTCTG |  |
| 10 | fwd | GGTTCAAAAAGAGGGATTTC |  |
|  | rev | GCTGGCAAGAATTCTGATAC |  |
| 11 | fwd | TGCAACTTTTGATGTAGGTG |  |
|  | rev | ACTGCCAACCTCATTTTATC |  |
| 12 | fwd | TGGTTCCTTTTGCTATGTTC |  |
|  | rev | TATCTCAGGAGCCAATAAGC |  |
| 13 | fwd | AGGGGTCTCTTATACCCTTG |  |
|  | rev | AGTAGAGATGGGGTTTCACC |  |
| 14 | fwd | CAGAGTCTCGCTCTATCACC |  |
|  | rev | GACTATCCTGGCTAACATGG |  |
| 15 | fwd | TGTCTCTCCTTGTTTCTTCC |  |
|  | rev | TGAGAAAGTCGTAGGGAAAC |  |
| 16 | fwd | GATGATCTCCATCTCCTGAC |  |
|  | rev | CGTTTTGAACAACCTCTTTC |  |
| 17 | fwd | CTGCCATTCAGAGTTGATGC |  |
|  | rev | GTCACTGAAAACCTGGCACA |  |
| 18 | fwd | GAATTCAAGGCTTCAGTGAG |  |
|  | rev | ACAAAAGCCAAGATTCATTC |  |
| GAPDH | fwd | AACCTGCCAAATATGATGAC |  |
|  | rev | AGGAAATGAGCTTGACAAAG |  |
| HPRT1 | fwd | AGATGTGATGAAGGAGATGG |  |
|  | rev | AATAGCTCTTCAGTCTGATAAAATC |  |
| 18S | fwd | GCAAAGCTGAAACTTAAAGG |  |
|  | rev | CGTTCGTTATCGGAATTAAC |  |

**References**

de la Mata M, Alonso CR, Kadener S, Fededa JP, Blaustein M, Pelisch F, Cramer P, Bentley D, Kornblihtt AR. 2003. A slow RNA polymerase II affects alternative splicing in vivo. *Mol Cell* **12**: 525-532.

Hoque M, Ji Z, Zheng D, Luo W, Li W, You B, Park JY, Yehia G, Tian B. 2013. Analysis of alternative cleavage and polyadenylation by 3[prime] region extraction and deep sequencing. *Nat Meth* **10**: 133-139.

Li W, You B, Hoque M, Zheng D, Luo W, Ji Z, Park JY, Gunderson SI, Kalsotra A, Manley JL et al. 2015. Systematic Profiling of Poly(A)+ Transcripts Modulated by Core 3’ End Processing and Splicing Factors Reveals Regulatory Rules of Alternative Cleavage and Polyadenylation. *PLoS Genet* **11**: e1005166.

Ray D, Kazan H, Cook KB, Weirauch MT, Najafabadi HS, Li X, Gueroussov S, Albu M, Zheng H, Yang A et al. 2013. A compendium of RNA-binding motifs for decoding gene regulation. *Nature* **499**: 172-177.

Wilusz JE, Spector DL. 2010. An unexpected ending: noncanonical 3' end processing mechanisms. *RNA* **16**: 259-266.
